# Effects of Recombinant α_1_-Microglobulin on Early Proteomic Response in Risk Organs after Exposure to ^177^Lu-Octreotate

**DOI:** 10.3390/ijms25137480

**Published:** 2024-07-08

**Authors:** Charlotte Ytterbrink, Emman Shubbar, Toshima Z. Parris, Britta Langen, Malin Druid, Emil Schüler, Sven-Erik Strand, Bo Åkerström, Magnus Gram, Khalil Helou, Eva Forssell-Aronsson

**Affiliations:** 1 Department of Medical Radiation Sciences, Institute of Clinical Sciences, Sahlgrenska Academy, University of Gothenburg, Sahlgrenska University Hospital, 413 45 Gothenburg, Sweden; charlotte.ytterbrink@vattenfall.com (C.Y.); emman.shubbar@gu.se (E.S.); malin.druid@regiondalarna.se (M.D.); 2 Sahlgrenska Center for Cancer Research, Sahlgrenska Academy, University of Gothenburg, 405 30 Gothenburg, Sweden; toshima.parris@oncology.gu.se (T.Z.P.); khalil.helou@oncology.gu.se (K.H.); 3 Department of Oncology, Institute of Clinical Sciences, Sahlgrenska Academy, University of Gothenburg, Sahlgrenska University Hospital, 413 45 Gothenburg, Sweden; 4 Section of Molecular Radiation Biology, Department of Radiation Oncology, University of Texas Southwestern Medical Center, Dallas, TX 75390, USA; britta.langen@utsouthwestern.edu; 5 Department of Radiation Physics, Division of Radiation Oncology, University of Texas M.D. Anderson Cancer Center, Houston, TX 77030, USA; eschueler@mdanderson.org; 6 Department of Clinical Sciences Lund, Oncology, Lund University, 221 00 Lund, Sweden; sven-erik.strand@med.lu.se; 7 Department of Clinical Sciences Lund, Infection Medicine, Lund University, 221 00 Lund, Sweden; bo.akerstrom@med.lu.se; 8 Department of Clinical Sciences Lund, Pediatrics, Lund University, 221 00 Lund, Sweden; magnus.gram@med.lu.se; 9 Department of Neonatology, Skåne University Hospital, 222 42 Lund, Sweden; 10 Biofilms—Research Center for Biointerfaces, Department of Biomedical Science, Faculty of Health and Society, Malmö University, 205 06 Malmö, Sweden; 11 Department of Medical Physics and Biomedical Engineering, Sahlgrenska University Hospital, 413 45 Gothenburg, Sweden

**Keywords:** A1M, antioxidant, proteomics, radio-protector, kidney, bone marrow, PRRT

## Abstract

Recombinant α_1_-microglobulin (A1M) is proposed as a protector during ^177^Lu-octreotate treatment of neuroendocrine tumors, which is currently limited by bone marrow and renal toxicity. Co-administration of ^177^Lu-octreotate and A1M could result in a more effective treatment by protecting healthy tissue, but the radioprotective action of A1M is not fully understood. The aim of this study was to examine the proteomic response of kidneys and bone marrow early after ^177^Lu-octreotate and/or A1M administration. Mice were injected with ^177^Lu-octreotate and/or A1M, while control mice received saline or A1M vehicle solution. Bone marrow, kidney medulla, and kidney cortex were sampled after 24 h or 7 d. The differential protein expression was analyzed with tandem mass spectrometry. The dosimetric estimation was based on ^177^Lu activity in the kidney. PHLDA3 was the most prominent radiation-responsive protein in kidney tissue. In general, no statistically significant difference in the expression of radiation-related proteins was observed between the irradiated groups. Most canonical pathways were identified in bone marrow from the ^177^Lu-octreotate+A1M group. Altogether, a tissue-dependent proteomic response followed exposure to ^177^Lu-octreotate alone or together with A1M. Combining ^177^Lu-octreotate with A1M did not inhibit the radiation-induced protein expression early after exposure, and late effects should be further studied.

## 1. Introduction

The radiopharmaceutical ^177^Lu-octreotate (Lutathera^®^, Advanced Accelerator Applications, Rueil-Malmaison, France) is used to treat patients with metastatic or progressive gastroenteropancreatic neuroendocrine tumors (GEP-NET). According to the European Medicines Agency (EMA), treatment with ^177^Lu-octreotate is given in a standardized manner with up to 4 cycles of 7.4 GBq at approximately 8 weeks apart [1]. However, the efficacy of ^177^Lu-octreotate-based treatment is limited by side effects on normal tissue, where bone marrow and the kidneys are the main dose-limiting organs. This standardized treatment schedule allows the risk of inducing toxicity to be kept very low, at the expense of the possibility of adapting the treatment to the individual patient [2]. A more personalized approached could be beneficial, since there are large inter-individual variations in both the renal absorbed dose from ^177^Lu-octreotate [3] and radiation sensitivity [4] within a patient group. By limiting the treatment schedule based on the most radiosensitive patients, a risk of undertreating a large proportion of patients arises. In contrast, increasing the total activity of ^177^Lu-octreotate might result in more effective treatment, but at the expense of increased treatment-related toxicity. However, the risk of normal tissue toxicity can be reduced by the use of radioprotective agents [5]. 

Following treatment with ^177^Lu-octreotate, protection of the kidneys is currently achieved by blocking the uptake of the radiopharmaceutical in the kidneys using positively charged amino acids, i.e., lysine and arginine [1]. Although these compounds are routinely used to reduce the absorbed dose to the kidneys, uptake of ^177^Lu-octreotate is only partly blocked, and side effects, like vomiting, are still common [6,7]. An alternative approach to protect the kidneys is to instead reduce harmful oxidative stress, induced by free radicals that are released during the interaction of ionizing radiation with biological tissues. Antioxidants are known to reduce oxidative stress in tissue, which makes them good candidates for radioprotection [8]. 

Recombinant α_1_-microglobulin (A1M) is an antioxidant and a potential candidate for the protection of normal tissue during ^177^Lu-octreotate treatment [9]. A1M has been described as a “radical sink”, meaning that by binding to the free radical and neutralizing the charge, A1M prevents further oxidation and thereby protects the tissue [10]. The distribution of A1M after i.v. injection in mice coincides, in the kidneys, with the distribution of similar somatostatin analogs used in ^177^Lu-octreotate treatment [11]. The potential radioprotective abilities of A1M have been studied in mice with promising results [12,13]. For example, co-infusion with A1M was shown to suppress the formation of DNA double-strand breaks and to inhibit the upregulation of apoptosis and stress-related genes in the kidney induced by ^177^Lu-octreotate. Furthermore, A1M also reduced kidney damage induced by ^177^Lu-octreotate on a long-term basis in mice, resulting in better overall survival compared with mice only receiving ^177^Lu-octreotate. Bone marrow cellularity and peripheral blood reticulocytes were preserved when mice injected with ^177^Lu-octreotate also received dual injections of A1M.

These results, together with the findings of our recent study, show that A1M does not interfere with the therapeutic effects of ^177^Lu-octreotate on NETs in tumor-bearing mice, thereby making A1M a promising candidate for kidney protection during ^177^Lu-octreotate treatment [14]. However, we still need to have a better understanding of the underlying mechanisms related to the protective antioxidant effects of A1M on tissue in order to further assess its potential therapeutic use. 

The complex puzzle of the interaction of radiation and tissue is not yet fully solved, and the need for radiobiology studies in radionuclide therapy is especially great [15,16]. Profiling of the proteomic and transcriptomic response to radiation has the potential to broaden our understanding of the mechanisms that lead to radiation-induced damage and can be a useful tool to identify biomarkers [17,18]. To the best of our knowledge, only a handful of studies have addressed the genomic or proteomic response in the kidneys after internal irradiation, including exposure to ^177^Lu-octreotate [19,20,21,22,23]. These studies show distinct differences in response between different absorbed doses, dose rates, and time after administration. The regulation patterns have been observed to be different between the kidney cortex and kidney medulla. In spite of such variations, exposure to ^177^Lu-octreotate yielded differences in the expression of transcripts and proteins in many cases, and potential biomarkers (Cdkn1a, Dbp, Lcn2, and Per2) could be proposed. These studies are the initial steps to chart which biological processes are initiated in healthy tissue during treatment with ^177^Lu-octreotate. However, to obtain a more complete picture of the response in risk organs, studies on the bone marrow need to be conducted. 

Presently, the genomic and proteomic response to the combination of radionuclide therapy and radioprotective agents is not well explored [16]. Profiling of the response can result in an improved understanding of the protective mechanisms and can contribute to the optimization of such treatment. To the best of our knowledge, no one has previously investigated A1M’s radioprotective abilities using an omics approach. The aim of the present study was to examine short-term differences in protein expression in bone marrow and kidneys after intravenous injection with ^177^Lu-octreotate and/or A1M, as well as A1M alone in mice.

## 2. Results

### 2.1. Absorbed Dose to Kidneys and Bone Marrow

The mean absorbed dose to the b one marrow, kidney inner medulla, kidney cortex, and total kidney was estimated from the biodistribution data for mice either injected with 150 MBq ^177^Lu-octreotate or injected with 150 MBq ^177^Lu-octreotate and A1M (5.0 mg/kg) (Table 1). The absorbed doses were similar for each tissue after 24 h irrespective of group, but somewhat lower at day 7 in the group that also received A1M.

### 2.2. Differentially Regulated Proteins, DRPs

**Figure 1:**
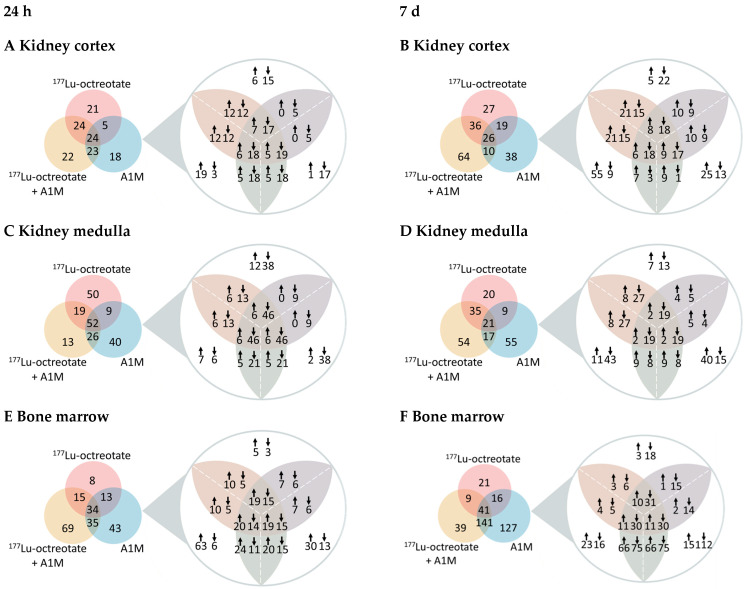
The total number of differentially regulated proteins in mouse tissues after exposure to ^177^Lu-octreoate, ^177^Lu-octreoate + A1M, or A1M only. Venn diagrams show unique and commonly expressed proteins with magnification showing the number of upregulated (↑) and downregulated (↓) proteins in (**A**) kidney cortex at 24 h, (**B**) kidney cortex at 7 d, (**C**) kidney medulla at 24 h, (**D**) kidney medulla at 7 d, (**E**) bone marrow at 24 h, and (**F**) bone marrow at 7 d.

Proteomics analysis revealed, at 24 h, 217 DRPs in bone marrow, 109 in the kidney cortex, and 157 in the kidney medulla (Figure 1A,C,E). At 7 d, 394 DRPs were identified in bone marrow, 194 in the kidney cortex, and 191 in the kidney medulla (Figure 1B,D,F). Fewer DRPs were found in bone marrow from the ^177^Lu-octreotate groups compared to the other groups (A1M and ^177^Lu-octreotate + A1M). In contrast, the highest number of DRPs in bone marrow was found in the A1M group at 7 d. About 40% of the DRPs in the A1M group after 7 d were unique, and about 43% were found in both the A1M and ^177^Lu-octreotate + A1M groups. In the kidney cortex, the highest number of DRPs was found in the ^177^Lu-octreotate + A1M group after 7 d. About 47% of the DRPs were unique to the combination group at that time point. Intriguingly, there was an overrepresentation of downregulated proteins (75% of the proteins were downregulated) in the kidney medulla. In both bone marrow and the kidney cortex, the number of DRPs was higher at the late time point.

Highly regulated DRPs, defined as those with |FC| > 90th percentile of that group, for each tissue type and time point, are listed in Table 2. The highest mean level of regulation was 6.9, and only 28 DRPs had an FC level above 4.0. Most of the DRPs with high regulation were found to be regulated in more than one group. Only three highly regulated DRPs were unique for the ^177^Lu-octreotate group, namely KRT82, KRT31, and KRT85 (all in kidney medulla), where KRT82 and KRT31 were also unique for the early time point. Seven highly expressed DRPs were unique for the combination group, five in the bone marrow (where CRYAB was unique for 24 h), and two in the kidney cortex (THRSP and EDRF1), both unique at 7 d. Among the highly expressed DRPs in the A1M group, ten DRPs were unique, with four in the kidney cortex, four in the kidney medulla, and two in bone marrow. ARHGAP23 was unique for the kidney medulla at 24 h. At day 7, all except one were time-unique: KV2A7, KV3A8, S100A9, and IGHG1 in the kidney cortex, LCN2 and CHIL3 in the kidney medulla, and IGHG1 and PTMS in bone marrow. 

**Figure 2:**
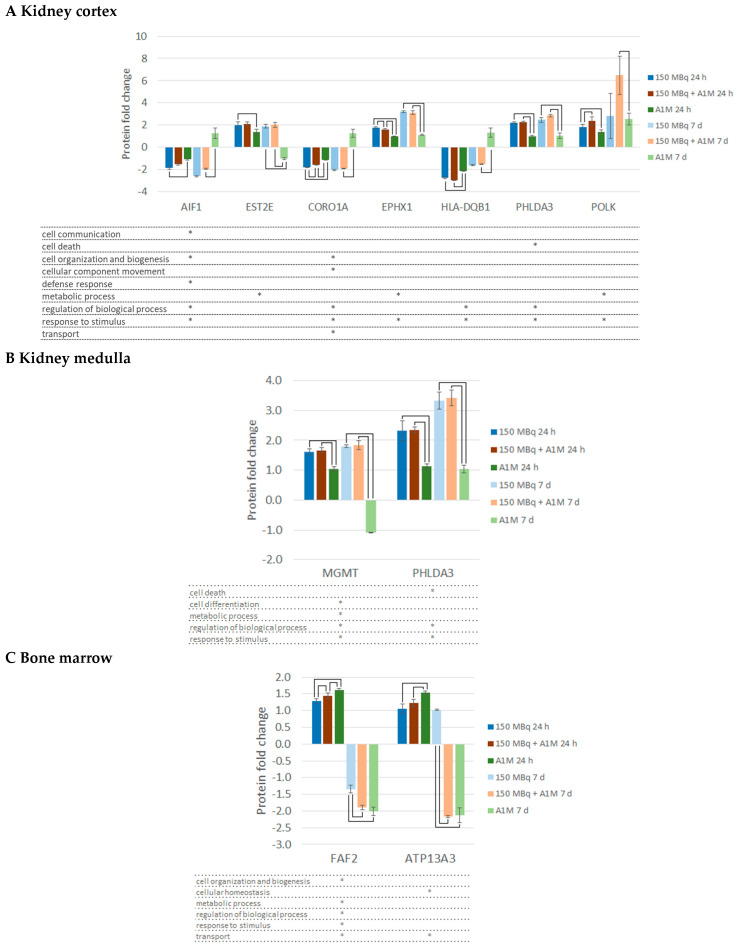
Proteins with significant regulation compared with control (|FC| ≥ 1.5) together with statistically significant differences in regulation between any of the groups (ANOVA, 5% FDR) at both time points in (**A**) kidney cortex, (**B**) kidney medulla, and (**C**) bone marrow. Error bars show the standard deviation, and brackets show statistically significant differences (*p* < 0.05). * indicates the biological function annotations for each protein, given by the Proteome Discoverer.

Results from the group comparison including all DRPs with a statistically significant difference between any of the groups at any of the time points (for each tissue type) are shown in Supplemental Tables S1–S3. In the kidney cortex, 42 proteins at 24 h and 76 proteins at 7 d showed statistically significant differences between any of the groups. At 24 h, most of these differences were found between the ^177^Lu-octreotate and the A1M group or/and between ^177^Lu-octreotate and combination group. At 7 d, most of the differences were found between the combination group and the A1M group. Moreover, statistically significant differences at both time points were found for seven proteins (AIF1, EST2E, COR1A, EPHX1, HLA-DQB1, PHLDA3, and POLK) (Figure 2A). In the kidney medulla, relatively few significant differences between the groups were found at 24 h (four proteins) and 7 d (nine proteins). Very few differences were found between the ^177^Lu-octreotate and combination groups. Statistically significant differences at both time points were found for only two of the proteins (MGMT and PHLDA3) (Figure 2B). In bone marrow, 3 proteins at 24 h and 117 proteins at 7 d were found to have a difference in regulation between any of the groups. Very few differences were found between the A1M group and the combination group. Statistically significant differences at both time points were found for only two proteins (FAF2 and AT.Z.P.13A3) (Figure 2C).

### 2.3. Canonical Pathway Analysis

IPA in silico canonical pathway analyses revealed that several of the regulated proteins were associated with a variety of pathways, most of them found in bone marrow in the ^177^Lu-octreotate + A1M group (Table 3). In bone marrow, the integrin linked kinase (ILK) signaling pathway was recurrently predicted as being activated in the ^177^Lu-octreotate + A1M group at both time points. In contrast, relatively few canonical pathways were identified in kidney tissue. In the kidney medulla, the 3-phosphoinositide biosynthesis and the superpathway of inositol phosphate compounds pathways were predicted as being inhibited at 24 h in the ^177^Lu-octreotate + A1M group as well as in the A1M-only group. In the kidney cortex, the estrogen receptor signaling pathway was found to be predicted as being inhibited in the ^177^Lu-octreotate + A1M group at 24 h and the Aryl hydrocarbon receptor signaling pathway was predicted to be inhibited in the ^177^Lu-octreotate group at 7 d.

### 2.4. Upstream Regulators

Upstream regulators were identified by IPA using the proteomics data (Table 4). In the kidney cortex, eight were identified in more than one group. The predicted state (activated or inhibited) of these common upstream regulators did not differ between the groups. Three of the eight upstream regulators were identified in the kidney cortex in all treatment groups at 24 h after injection (SIRT1 (activated), STAT1 (inhibited), and TRIM24 (activated)). STAT1 and TRIM24 were also identified in the kidney medulla at 24 h after injection of ^177^Lu-octreotate, with the same predicted activated state. None of the identified upstream regulators in the kidney medulla were found in more than one group. In bone marrow, nine upstream regulators were identified in more than one treatment group. KDM5A was identified in all groups at 7 d after injection and was predicted to be activated in the ^177^Lu-octreotate group and inhibited in the other groups. A complete list of the identified upstream regulators is shown in Supplemental Table S4.

### 2.5. Toxicity Functions

To predict any potential toxicity in investigated tissues, in silico analyses with IPA’s toxicity function were performed. All predicted nephrotoxicity functions (with calculated z-scores) in the dataset are shown in Table 5. Results from predicted hepatotoxicity or cardiovascular toxicity are shown in Supplemental Table S5. Based on the regulated proteins in the kidney cortex, the simulation found a relation to the nephritis function in the combination group at 7 d. In the kidney medulla, functions related to glomerulosclerosis and cell death were found 24 h after the injection of ^177^Lu-octreatate. Furthermore, at 7 d, the cell viability function was found in the combination group, and a relation to cell death function was observed in the A1M only group. None of the related functions were predicted to be activated (z ≥ 2.0) or inhibited (z ≤ −2.0) in any of the groups or time points in any of the kidney tissues.

## 3. Discussion

In the present study, we examined differences in proteomic response in risk organs after exposure to ^177^Lu-octreotate alone or in combination with the potential radioprotector A1M, as well as with A1M alone. The radiation-induced response on the proteome was observed in the kidney cortex and medulla 24 h and 7 d after administration. The expression of these radiation-related proteins did not generally differ between the ^177^Lu-octreotate and the ^177^Lu-octreotate + A1M groups. IPA in silico analyses of the protein dataset identified canonical pathways and upstream regulators in all investigated tissues and toxicity functions in kidney tissue.

The proportion of group-common DRPs (proteins regulated in all groups) was relatively high in all tissues and time points. A more treatment specific response was expected, and the observed similarities between all groups are surprising. In kidney tissue, about 25–40% of the DRPs were unique for the ^177^Lu-octreotate group. In general, very few DRPs were common between only the ^177^Lu-octreotate group and the A1M group. Thus, the response in both groups receiving ^177^Lu-octreotate showed higher similarities, which could be expected, as the high amount of ^177^Lu-octreotate (150 MBq) was chosen since it is known to result in high nephrotoxicity. Most common DRPs in kidney tissue had changes in the same direction, also between the ^177^Lu-octreotate group and the A1M group. In bone marrow, the number of DRPs with changes in the opposite direction was somewhat higher between these groups. In the kidney medulla, an overrepresentation of downregulated DRPs was observed in the ^177^Lu-octreotate group, while this was not observed in the kidney cortex. In bone marrow, a lower fraction of unique DRPs was observed in the ^177^Lu-octreotate, and the total number of DRPs was lower in the ^177^Lu-octreotate group compared to the other groups. This indicates a lesser proteomic response to ^177^Lu-octreotate in bone marrow. The opposite was observed in the proteomic response to A1M exposure, especially at 7 d, when the highest number of DRPs in bone marrow was found in the A1M group. Altogether, the number of unique and commonly expressed proteins indicate a better agreement in protein regulation between the ^177^Lu-octreotate group and the combination group in the kidney. In bone marrow, more similar protein regulation was observed between the A1M group and the combination group, and the effect of A1M seems to increase with time.

^177^Lu-octreotate exposure resulted in a few unique highly regulated DRPs (|FC| > 90th percentile), all of them keratin proteins. There are many keratin protein types, half of which are involved in the hair follicle, while the rest are involved in other processes. Non-hair keratin proteins are important for integrity of epithelial cells and tissue, and are also involved in protection from, e.g., stress and apoptosis, which are known radiation-induced effects. Non-hair keratin proteins have previously been associated with epithelial cell injury in mouse kidney [24,25]. Other non-hair keratin proteins, such as KRT71 and KRT16, were also found among the group with common highly regulated DRPs in the kidney cortex and medulla and bone marrow. Regarding hair keratins, we cannot, however, rule out that there have been hair contamination of any sample during tissue handling. Many of the highly regulated DRPs unique for the A1M group are associated with immune and inflammatory responses, e.g., neutrophil gelatinase-associated lipocalin (LCN2) and protein S100-A9 (S100A9), upregulated in the kidney medulla and kidney cortex, respectively [26]. LCN2 has previously been associated with acute kidney injury and is an established biomarker for kidney damage [27]. The gene expression of LCN2 (also known as NGAL) has previously been studied in kidney tissue 6 weeks after the injection of A1M and was not found to be significantly regulated compared with the control [13]. 

Among the significantly regulated proteins, Pleckstrin homology-like domain family A member 3 (PHLDA3), a known apoptotic related protein, was found to be recurrently upregulated in both the kidney medulla and cortex in the irradiated groups. PHLDA3 has not only been suggested to be a radiation-responsive gene [28], but its transcript has also been found to be regulated in mouse kidneys after exposure to ^177^Lu-octreotate [19]. Furthermore, we have also demonstrated other transcripts of recurrent DRPs observed in the present study in a previous experiment: *Ephx1* encoding Epoxide hydrolase 1 (EPHX1) and *H2-Ab1* encoding histocompatibility 2, class II antigen A, beta-1 (HLA-DQB1) [19]. In the present study, EPHX1 was upregulated in the kidney cortex at both time points, with a higher regulation (statistically significant at 24 h) in the ^177^Lu-octreotate group compared to the other groups. HLA-DQB1 was downregulated at both time points after injection of ^177^Lu-octreotate or ^177^Lu-octreotate + A1M, with no statistically significant difference in the regulation between these groups.

Other DRPs, in addition to PHLDA3, that are known to be encoded by radiation-responsive genes, include Bcl-2-binding component 3 (BBC3, also known as P53 upregulated modulator of apoptosis (PUMA)), apoptosis regulator BAX (BAX), serum amyloid A-1 protein (SAA1), and haptoglobin (HP) [28,29,30]. BBC3 and BAX both belong to the B cell CLL/lymphoma-2 family (BCL2), a family of anti- and pro-apoptotic proteins, which regulate the mitochondrial pathway of apoptosis [31]. BAX is one of the effector proteins that activates the mitochondrial pathway of apoptosis. BBC3 is a so-called sensitizer, i.e., it is involved in indirect initiation of apoptosis by facilitating the activation of effector proteins [31]. SAA proteins are involved in immunological responses during inflammation (a known response to irradiation) and SAA1 has been proposed as a biodosimetry marker that is activated shortly after radiation exposure [32]. 

In this study, BBC3 and SAA1 (and SAA2) were regulated in the kidney cortex at 24 h, and BAX was regulated in both the kidney cortex and medulla at 7 d. However, the regulation of PHLDA3, BAX, and BBC3 in the ^177^Lu-octreotate group was not significantly different from that in the ^177^Lu-octreotate + A1M group at any of the time points. SAA1 and SAA2 were upregulated in cortex in the ^177^Lu-octreotate + A1M and A1M group, but not in the ^177^Lu-octreotate group. The observed expression pattern of SAA1 and SAA2 could potentially be interpreted as an inflammatory response induced as a response to A1M exposure. This is surprising since A1M homologs purified from human and animal plasma and urine have been described to have immunologic, but mostly immunosuppressive and anti-inflammatory, properties [33]. The regulation of HP was significantly higher in the combination group at 24 h in the kidney cortex. At the same time point, HP was also upregulated in the combination group in the medulla, although not significantly higher than in the other groups. HP has previously been found to be over expressed in bone marrow after irradiation [30,34,35]. Nevertheless, since HP was only found to be upregulated in the combination group and not in the irradiation only group, the changes in the HP level are not likely to be a response to irradiation only, but rather a response to a combination of radiation and A1M in irradiated tissue. A1M and HP both play an important role in the defense against toxic levels of hemoglobin (Hb) and heme [36,37]. During hemolysis, heme and Hb are released from ruptured red blood cells and accumulate in the kidney. HP is known to capture Hb during hemolysis, and the resulting Hb/HP complex is cleared in the liver by the macrophage CD163 scavenger receptor [37]. A1M can minimize damage from hemolysis by binding to heme, and it reduces extracellular Hb levels [36]. Thus, it could be speculated that the presence of A1M in the tissue after treatment contributes to further activation of defense mechanisms against free Hb and heme via the upregulation of HP. In this study, HP was not found to be regulated in the bone marrow, but higher levels of HP were found in kidney tissue. 

The radiation response in bone marrow was less prominent compared to the kidneys. The majority of the DRPs were found in the combination or A1M group, with only a few DRPs in the ^177^Lu-octreotate-only group. One of the DRPs in bone marrow, alpha-1-antitrypsin 1-1 (SERPINA1A), has a close relation to alpha-1-antitrypsin 1-3 (SERPINA1C) [27]. SERPINA1C has previously been found to be upregulated in mouse bone marrow 24 h after γ-irradiation with an absorbed dose of 4 Gy [30,34]. In the present study, SERPINA1A was found to be upregulated in bone marrow at 24 h in the ^177^Lu-octreotate and in the combination group, although the regulation was not statistically significant different between any of the groups, including the A1M group. Compared to the kidney, the indistinct radiation response shown in bone marrow is unclear, but it could partly be explained by organ-dependent radiation sensitivity. The bone marrow is more sensitive to radiation than the kidneys and severely damaged bone marrow cells are less likely to survive, which could give a lesser proteomic response compared to repairable surviving kidney cells. Interestingly, a stronger response to A1M was observed in bone marrow compared to kidney tissue. Many of the DRPs were unique for the A1M group, and the total number of DRPs in bone marrow drastically increased with time after the A1M injection. It may be speculated that the previously reported interactions between A1M and blood cells, i.e., binding, as well as effects on red blood cells stability and immune and inflammatory responses, could explain the effects on protein expression seen in the present study [33,38,39,40].

The in silico canonical pathway analyses showed a difference in the number of identified pathways between the tissues; only a few pathways were identified in the kidney, but several were identified in bone marrow. ILK signaling was the most commonly associated pathway in the bone marrow dataset. ILK is a multifunctional protein that is involved in cellular functions, like cell migration, differentiation, survival, senescence, and division [41]. The simulation predicted that ILK would be activated in the combination group at both time points and inhibited in the A1M group at 24 h as well as in the ^177^Lu-octreotate group at 7 d. Other pathways identified in more than one group or time point in the bone marrow dataset included calcium signaling (activated at 7 d in the combination group and the A1M group), regulation of actin-based motility by Rho (activated in the combination group at both times), and signaling by Rho family GTPases (activated in the combination group at both times). For these pathways, as well as for ILK signaling, ACTA1 and ACTC1 (belonging to the actin gene family) are involved proteins found in the dataset. Furthermore, several members of the MYH or MYL gene families are involved in these pathways and commonly found in the dataset. 

The in silico upstream regulator analyses showed that a handful of irradiation-associated molecules were affected in kidney tissue. In the kidney cortex, SIRT1 was identified as a predicted activated upstream regulator in all groups at 24 h. SIRT1 is a nicotinamide adenine dinucleotide (NAD)-dependent deacetylase that participates in several cellular functions, including the response to DNA damage, the cell cycle, metabolism, apoptosis, and autophagy. SIRT1 has been found to be involved in renal pathologies, like metabolic kidney diseases and acute kidney damage [42]. STAT1, a promotor of both apoptotic and non-apoptotic cell death [43], was predicted to be inhibited at 24 h in all groups in the cortex as well as in the ^177^Lu-octreotate group in the medulla. These findings are in agreement with our previous study of microRNA (miRNA) expression analysis following treatment with ^177^Lu-octreotate, where STAT1 was identified as a predicted inhibited upstream regulator [22]. Furthermore, Ifnar, involved in the modification of STAT1 by Janus protein kinase-activated phosphorylation, was identified as a predicted inhibited upstream regulator in the present study. Only one identified upstream regulator, i.e., mir-21, was an miRNA. In a study investigating miRNAs as a urinary biomarkers for radiation-induced kidney damage, mir-21 was presented as a promising candidate [44]. In the present study, mir-21 was a commonly identified upstream regulator predicated to be activated in the cortex at 24 h in the A1M group and at 7 d in the combination group, as well as in the medulla at 24 h in the ^177^Lu-octreoate group. Furthermore, mir-21 is a known radiation-responsive miRNA, and activation of mir-21 in mouse kidneys after ^177^Lu-octreotate exposure has also been observed in our previous study [22]. It is unclear why activation was obtained in the A1M group. In our previous study, we also found that the cytokine IFNG was predicted to be an inhibited upstream regulator [22]. This corresponds well with the results from the present study, where inhibition was found at 24 h in the cortex (combination group and A1M group) and medulla (^177^Lu-octreotate group). IFNG was also identified as one of the primary upstream regulators in one of our previous studies investigating transcriptional effects in kidney tissue after ^177^Lu-octreotate administration in mice [21]. Taken together, radiation-associated upstream regulators were identified in kidney tissue. No clear differences between ^177^Lu-octreotate and the combination groups were found in predicted activation state. Furthermore, some of these upstream regulators were also identified in mice that had received only A1M. Based on these results, some of the predicted activation or inhibition of these upstream regulators might be related to the A1M exposure. This finding, however, needs to be carefully investigated in future studies. It should be noted that the IPA analyses are simulations based on the expressions of the proteins in the dataset and should be considered as predictions of up/down-stream effects. Further studies are needed to confirm the predicted upstream regulators, as well as the affected canonical pathways and toxicity functions identified in this study.

The IPA in silico toxicity function analyses identified proteins in the dataset that are connected to nephrotoxicity. Taken together, these results predict relations to inflammation, glomerular injury, and cell death in the kidney after the injection of ^177^Lu-octreotate with or without A1M. Based on the regulation of the proteins, the analyses could not predict if the functions were inhibited or activated, which limits our ability to draw conclusions concerning any induced or prevented kidney toxicity. No histological evaluation or other analysis methods (other than IPA) were used to assess toxicity in the kidneys, since it is not likely that the radiation inflicted any histopathologically detectible changes in the kidneys at such early time points (24 h and 7 day). However, other toxicity assessments parameters should be considered in future studies, also including investigations at later time points.

The present study was performed on female mice only, since we wanted to include mice of the same sex in all groups to avoid potential differences in the response due to sex. Female mice were chosen to enable the comparison of results with corresponding studies on tumor-bearing mice, which are performed on female mice due to their much higher tumor take of neuroendocrine tumors compared to male mice. In the present study the group size was 10, which is somewhat higher than we usually use. The reason was to receive better statistics, since some previous studies on radiation-induced gene and protein expression gave results with large interindividual variations. It should be noted that no mice died during the study.

The absorbed dose calculations in this study do not include contributions from photons and cross doses from other surrounding tissues. Thus, the absorbed doses are somewhat underestimated. However, ^177^Lu has a low photon yield and the emitted electrons have a short mean range (0.67 mm in water). Taking this into account, together with interindividual differences, the dosimetric estimations should be reasonable.

To the best of our knowledge, this is the first investigation of the proteomic response in the kidney and/or bone marrow at these early time points after the injection of ^177^Lu-octrotate. Our findings show that regulation of radiation-responsive proteins can be detected early after exposure to ^177^Lu-octrotate in kidney tissue, which is otherwise a late responding organ when it comes to functional damage [21]. These proteins are related to processes, such as apoptosis and inflammation, which can result in damage to and loss of function of the organ. No clear indication of altered regulation in these radiation-responsive proteins was shown when ^177^Lu-octreotate was co-administrated with A1M, indicating that A1M does not mitigate the radiation response in kidney tissue. 

The regulation of radiation-responsive proteins was lower in bone marrow, which is surprising since it is otherwise an early radiation-responding organ. The response to A1M was more profound in bone marrow compared with the kidney, especially at the later time point (7 d). 

The reasons why we did not demonstrate a large protective effect of A1M are not obviously explained. We chose doses of ^177^Lu-octreotate and A1M that had previously showed various effects in similar studies. One factor could be the choice of evaluation time after injection, since the expression of proteins after intervention should vary with time. In the present study, we chose both one early and one somewhat later time point, and we hoped that we could find interesting responses. It is also possible that one injection of 5 mg/kg A1M is not enough to achieve radioprotection of the kidneys at the high activity amount administered, 150 MBq ^177^Lu-octrotate, and also since the irradiation will continue for a long time period due to accumulation in kidneys. Studies on combination treatment with ^177^Lu-octreotate and multiple injections of A1M are currently ongoing in our research group. Furthermore, the long-term effects of the combination treatment with ^177^Lu-octreotate and A1M are still unknown and should be followed over time, preferably by using biomarkers for kidney and bone marrow damage, measured in urine and/or blood.

## 4. Materials and Methods

### 4.1. Radiopharmaceutical

LuMark^® 177^Lu chloride and peptide were obtained from the Nuclear Research and Consultancy Group (IDB Holland, Baarle-Nassau, The Netherlands). Radiolabeling was conducted according to the manufacturer’s instructions. Instant thin-layer chromatography (ITLC), using Whatman™ chromatography paper (3 mm, GE Healthcare UK Limited, Amersham, Great Britain) and 0.1 M sodium citrate (Labservice AB, Sundsvall, Sweden) showed that the amount of peptide-bound ^177^Lu was higher than 99%. Syringes containing the desired ^177^Lu activity (in 0.1 mL) were prepared from the ^177^Lu-octreotate solution and measured according to a previously published method [14]. 

### 4.2. Recombinant α_1_-Microglobulin (A1M)

Human recombinant A1M (modified variant A1M-035 [45], concentration of 5.9 mg/mL) and rA1M vehicle solution containing sterile endotoxin-free 10 mM Na_3_PO_4_ (pH 7.4), 0.15 M NaCl, and 12 mM histidine were supplied by A1M Pharma (Lund, Sweden) (new name: Guard Therapeutics International AB, Stockholm, Sweden). rA1M was diluted to a concentration of 1.1 mg/mL and dosed based on each individual mouse body weight, to a final dose of 5.0 mg/kg. The abbreviation A1M will be used for all further description of rA1M in this paper.

### 4.3. Animal Experiments

A total number of 50 6–12 week old female C57/6N mice (Charles River Laboratories International, Inc., Salzfeld, Germany) were included in this study and divided into 5 groups of 10. Three groups of mice received two i.v. injections each, with (a) 150 MBq ^177^Lu-octreotate and phosphate buffered saline solution (PBS), (b) 5 mg/kg A1M and PBS, or (c) 150 MBq ^177^Lu-octreotate and 5 mg/kg A1M. As controls, mice in two sham-treated groups received two injections each with either PBS or PBS and A1M vehicle solution (see details in Section 4.2 Recombinant α_1_-Microglobulin (A1M)). Half the number of mice in the five groups were killed 24 h after injection and the other mice were killed 7 d after injection. The mice were killed by cardiac puncture under anesthesia with i.p.-administered sodium pentobarbital (0.25 mg, APL, Stockholm, Sweden). At the time of death, the femur and one of the kidneys were collected from the animals, flash-frozen with liquid nitrogen, and stored at −80 °C until further analysis. Bone marrow was separated from the femur, while the kidney medulla and kidney cortex were excised from the frozen kidneys using a scalpel. During the experiment, the mice were kept in ventilated cages under standard laboratory conditions and were given water and food ad libitum. The study was approved by the Ethics Committee for Animal Research in Gothenburg, Sweden (no. 146-2015).

### 4.4. Radioactivity Measurements

^177^Lu activity was measured in kidneys fixed in formaldehyde using a gamma counter (2480 Wizard Automatic Gamma Counter, PerkinElmer, Waltham, MA, USA). Measurements were corrected for dead-time losses and background radiation. The measured activity in the samples was corrected for radioactive decay to time of injection. The gamma counter was cross-calibrated with a well-type ionization chamber (CRC-15R, Capintec, Ramsey, NJ, USA) used to determine the activity of ^177^Lu in the syringes prior to injection. Due to the limited volume of the bone marrow samples, no radioactivity measurement was feasible.

### 4.5. Absorbed Dose Calculation

#### 4.5.1. Bone Marrow

The mean absorbed dose to the bone marrow was calculated according to the MIRD formalism [46] as:

(1) $$D\left(r_{BM},T_{D}\right)=\frac{\tilde{A}\left(r_{BM},T_{D}\right)}{M\left(r_{BM}\right)}\sum_{i}E_{i}Y_{i}\varphi (r_{BM}\leftarrow r_{BM})$$

where $\tilde{A}(r_{BM},T_{D})$ is the time-integrated activity over the time period $T_{D}$ in the bone marrow $r_{BM}$, and $M(r_{BM})$ is the bone marrow mass. $Y_{i}$ is the yield of radiation i with energy $E_{i}$, and $\sum_{i}E_{i}Y_{i}$ was set to be 148 keV [47], only considering electrons. The self-absorbed fraction, $\varphi \left(r_{BM}\leftarrow r_{BM}\right)$, of the electrons emitted in the target organ was set to 0.738 [48], and the cross-absorbed fractions from the surrounding tissues were set to 0. The time-integrated activity per organ weight was calculated based on data on the activity concentration from previous biodistribution studies [49]. Integrations were performed with the trapezoidal rule, and the activity at t = 0 was assumed to be zero. The mean absorbed dose was calculated with the assumption of homogeneous activity distribution in the bone marrow.

#### 4.5.2. Kidneys

The mean absorbed dose to the kidneys was calculated using *S* values according to the MIRD formalism [46] as:

(2) $$D\left(r_{T},T_{D}\right)=\tilde{A}\left(r_{S},T_{D}\right)\;S\left(r_{T}\leftarrow r_{S}\right),$$

where $S(r_{T}\leftarrow r_{S})$ is the absorbed dose rate per unit activity and $\tilde{A}\left(r_{S},T_{D}\right)$ is the time-integrated activity. The absorbed dose was calculated for the inner medulla, cortex, and whole kidney using Monte Carlo-derived *S* values [49]. The time-integrated activity was calculated based on data from previous biodistribution studies [48] (activity concentration at 0.25 h to 3 d after injection) as well as the activity measurements in the present study (activity concentration at 24 h and 7 d). The trapezoidal rule was used for the integration, and the activity at t = 0 was assumed to be zero.

### 4.6. Proteomics

Samples of bone marrow, kidney medulla, and kidney cortex were selected for protein analysis. Individual samples from 6/10 mice in each treatment group (3/5 in each study group) as well as pooled samples from 10/10 individuals from each sham-treated control group (5/5 in each study group) were analyzed. The proteomic analysis was performed at the Proteomics Core Facility at Sahlgrenska Academy, University of Gothenburg, Sweden. The protein data were uploaded to the Proteomic identifications database (PRIDE) (project accession: PXD029937).

#### 4.6.1. Sample Preparation and Digestion

Samples were homogenized using a FastPrep^®^-24 instrument (MP Biomedicals, Santa Ana, CA, USA) with Lysing Matrix D (1/3 of the original number of beads) for five repeated cycles (speed 6.5 m/s, 40 s/cycle) in 100 µL of the buffer containing 2% sodium dodecyl sulfate and 50 mM triethylammonium bicarbonate (TEAB). Samples were centrifuged at 16,000× *g* for 10 min, and the supernatants were transferred to clean tubes. The lysis tubes were washed with 100 µL of the lysis buffer, centrifuged at 16,000× *g* for 10 min, and the supernatants were combined with the corresponding lysate from the previous step. Protein concentration in the combined lysates was determined using Pierce™ BCA Protein Assay Kit (Thermo Scientific, Waltham, MA, USA) and a Benchmark™ Plus microplate reader (BIO-RAD, Hercules, CA, USA) with bovine serum albumin (BSA) solutions as standards. Two different representative reference pools were prepared from an aliquot of all samples from the medulla and cortex or bone marrow. 

#### 4.6.2. Tryptic Digestion and Tandem Mass Tag (TMT) Labelling

The samples and reference samples were digested with trypsin using the filter-aided sample preparation (FASP) method [50]. Briefly, 30 µg from each sample and the references were reduced with 100 mM dithiothreitol at 60 °C for 30 min, transferred to 30 kDa MWCO Pall Nanosep centrifugation filters (Sigma-Aldrich, Saint Louis, MO, USA), and washed several times with 8 M urea and once with digestion buffer prior to alkylation with 10 mM methyl methanethiosulfonate in digestion buffer for 20 min. Digestion was performed in 50 mM TEAB and 0.5% sodium deoxycholate (SDC) buffer at 37 °C with the addition of 0.3 µg Pierce MS grade Trypsin (Thermo Scientific, Waltham, MA, USA), and the solution was incubated overnight. An additional portion of trypsin was added and incubated for another four hours. Peptides were collected by centrifugation. The samples in each study were divided into six TMT sets. All sets included a reference pool to be able to compare the samples within a set as well as between sets, from the same tissue or between medulla and cortex. Peptides were labelled using TMT 11-plex isobaric mass tagging reagents (Thermo Scientific, Waltham, MA, USA) according to the manufacturer’s instructions, and SDC was removed by acidification with 10% TFA. The TMT sets were desalted before being pre-fractionated into 40 fractions with basic reversed-phase chromatography (bRP-LC) using a Dionex Ultimate 3000 UPLC system (Thermo Scientific, Waltham, MA, USA). Peptide separation was performed using a reversed-phase XBridge BEH C18 column (3.5 μm, 3.0 × 150 mm, Waters Corporation, Milford, MA, USA) and a linear gradient from 3% to 45% acetonitrile in 10 mM ammonium formate buffer at pH 10.00 over 17 min followed by an increase to 90% acetonitrile over 5 min. The fractions were concatenated into 20 fractions, dried, and reconstituted in 3% acetonitrile and 0.2% formic acid. 

#### 4.6.3. LC-MS/MS Analysis

The fractions were analyzed on an Orbitrap Fusion Lumos Tribrid mass spectrometer interfaced with an Easy-nLC1200 liquid chromatography system (both Thermo Scientific, Waltham, MA, USA). Peptides were trapped on an Acclaim Pepmap 100 C18 trap column (100 μm × 2 cm, particle size 5 μm, Thermo Scientific, Waltham, MA, USA) and separated on an in-house packed analytical column (75 μm × 45 cm, particle size 3 μm, Reprosil-Pur C18, Dr. Maisch) using a linear gradient from 5% to 33% B over 77 min followed by an increase to 100% B for 3 min, and 100% B for 10 min at a flow of 300 nL/min. Solvent A was 0.2% formic acid in water and solvent B was 80% acetonitrile and 0.2% formic acid. MS scans were performed at 120,000 resolution, with an *m*/*z* range 375–1375; MS/MS analysis was performed in a data-dependent manner, with top speed cycle of 3 s for the most intense doubly or multiply charged precursor ions. The most intense precursors were fragmented in MS2 by collision-induced dissociation (CID) at 35 eV collision energy with a maximum injection time of 50 ms and then detected in the ion trap, followed by multi-notch (simultaneous) isolation of the top 10 MS2 fragment ions, with *m*/*z* 400–1400; fragments were selected for fragmentation (MS3) by higher-energy collision dissociation (HCD) at 65% and detection in the Orbitrap at 50 000 resolution, with *m*/*z* 100–500. Precursors were isolated in the quadrupole with a 0.7 *m*/*z* isolation window, and dynamic exclusion within 10 ppm for 45 s was used for *m*/*z* values already selected for fragmentation.

### 4.7. Proteomic Data Analysis 

The data files for the sets from the same tissue were merged for identification and relative quantification using Proteome Discoverer version 2.4 (Thermo Scientific, Waltham, MA, USA). The search was carried out against the Mouse Swissprot Database version June 2019 (Swiss Institute of Bioinformatics, Lausanne, Switzerland) using Mascot 2.5 (Matrix Science, Chicago, IL, USA) as a search engine with a precursor mass tolerance of 5 ppm and fragment mass tolerance of 0.6 Da. Tryptic peptides were accepted with zero missed cleavage and variable modifications of methionine oxidation, and fixed cysteine alkylation, TMT-label modifications of N-terminal and lysine were selected. The references were used as denominators and for calculation of the ratios. The Percolator algorithm in Mascot was used for the validation of identified proteins, and the quantified proteins were filtered at 1% FDR and grouped by sharing the same sequences to minimize redundancy. TMT reporter ions were identified in the MS3 HCD spectra with 3 mmu mass tolerance, and the TMT reporter intensity values for each sample were normalized on the total peptide amount. Only peptides unique for a given protein were considered for quantification.

### 4.8. Analysis of Protein Regulation

Protein regulation (fold-change, FC) was calculated by dividing the abundance of the protein in the treatment groups by the abundance of the corresponding control groups. Differently regulated proteins (DRPs) were defined as geometric mean |FC| ≥ 1.5, where FC ≥ 1.5 means upregulation and FC ≤ −1.5 means down regulation compared with control. Calculation of FC and statistical analyses was performed using Perseus version 1.6.10.50 (http://www.perseus-framework.org (accessed on 19 February 2020)). Annotations to biological functions were given by the Proteome Discoverer. The different time points and tissues were analyzed separately. The differences between the treatment groups were determined by performing one-way ANOVA followed by pairwise comparison with Welch’s test. For the statistical analyses, only proteins with a geometric mean |FC| ≥ 1.5 in at least one treatment group were considered. All statistical analyses were permutation based with 5% FDR.

In silico analyses of canonical pathways, upstream regulators and toxicity function analyses were performed based on regulated proteins using the Ingenuity Pathway Analysis (IPA) software version 51963813 (Qiagen, Hilden, Germany). The IPA software’s in silico toxicity function identifies biological functions related to hepatotoxicity, nephrotoxicity, or cardiovascular toxicity. In this study toxicity function analyses were performed on protein data from kidney and bone marrow. A Fisher’s exact test p-value cutoff of 0.05 was used for all IPA analyses. The IPA analyses only considered molecules and/or relationships found in mice and humans. Predicted activation state was determined using z-score, where z ≤ −2.0 indicates inhibition and z ≥ 2.0 indicates activation. For the upstream regulator and toxicity functions analyses, a bias-corrected z-score was used with the exceptions for cases with strong bias when activation z-score was used, according to the manufacturer’s recommendations.

## 5. Conclusions

Exposure of mice to ^177^Lu-octreotate and/or the proposed radioprotector A1M resulted in a tissue-specific proteomic response 24 h and 7 d after administration in kidney and bone marrow, the two major risk organs in ^177^Lu-octreotate therapy. Early after ^177^Lu-octreotate administration, regulatory effects were found for previously observed radiation-responsive proteins that are related to cell death and inflammation. In the kidney, PHLDA3 was the most recurrent regulated protein and has pro-apoptotic effects. Co-administration of A1M and ^177^Lu-octreotate did not in general alter the regulation of the observed radiation-responsive proteins. Thus, no clear reduction or inhibition of radiation-induced response in risk organs was observed when A1M was administered with ^177^Lu-octreotate. After a single injection of A1M, signs of immune and inflammatory response were observed, and potential functional effects of these observations remain to be elucidated. Furthermore, the potential long-term effects of co-administration of ^177^Lu-octreotate and A1M are still unknown. This knowledge is needed before concluding the potential radioprotective usefulness of A1M in ^177^Lu-octreotate treatment. 

## Figures and Tables

**Table 1:** The mean absorbed dose to the bone marrow, kidney inner medulla, kidney cortex, and total kidney after injection of 150 MBq ^177^Lu-octreotate or 150 MBq ^177^Lu-octreotate and A1M (5.0 mg/kg).

	Mean Absorbed Dose (Gy)			
	^177^Lu-Octreotate		^177^Lu-Octreotate + A1M	
Time after Injection	24 h	7 d	24 h	7 d
Bone marrow	6.0 Gy	21 Gy	5.8 Gy	19 Gy
Kidney inner medulla	28 Gy	73 Gy	27 Gy	66 Gy
Kidney cortex	25 Gy	64 Gy	24 Gy	58 Gy
Total kidney	25 Gy	66 Gy	25 Gy	59 Gy

**Table 2:** Fold-change (FC) values for the highest regulated proteins (|FC| above the 90th percentile within each group and tissue type) in mouse kidney cortex, kidney medulla, and bone marrow at 24 h and 7 d after injection of ^177^Lu-octreotate (^177^Lu), ^177^Lu-octreotate with A1M (^177^Lu + A1M), or with A1M alone. **Bold** indicates group-unique proteins (only regulated in that treatment group), and * indicates also that the protein was unique for that time point. FC ≥ 1.5 means upregulation (blue) and FC ≤ −1.5 means downregulation (orange).

	24 h						7 d					
	^177^Lu		^177^Lu + A1M		A1M		^177^Lu		^177^Lu + A1M		A1M	
Kidney cortex	BCHE	−5.3	BCHE	−4.2	SAA2	4.7	SNRK	−4.3	POLK	6.5	KV2A7 *	3.5
	TGT.Z.P.1	−3.8	SAA2	3.5	PLIN1	−3.7	GLE1	−4.2	CMA1	5.8	SNRK	−3.1
	AQP4	3.1	HP	3.5	BCHE	−3.1	CACTIN	−3.7	THRSP *	5.1	HP	2.9
	SDSL	−3.1	TGT.Z.P.1	−3.3	TGT.Z.P.1	−2.9	EPHX1	3.2	SNRK	−3.8	KV3A8 *	2.9
	LSP1	−3.0	SDSL	−3.1	C.Y.P2A4	−2.8	HDC	−3.1	CACTIN	−3.2	AQP4	2.8
	GLE1	−2.6	H2-AB1	−3.0	SAA1	2.7	PLCD4	2.8	EPHX1	3.1	GCAB	2.8
	H2-AB1	−2.7	KRT71	2.6	SLC22A13	−2.5	POLK	2.8	PLCD4	2.9	S100A9 *	2.6
	CACTIN	−2.4	C.Y.P2A4	−2.5			PNPLA2	−2.6	EDRF1 *	2.9	POLK	2.6
			SAA1	2.4			AIF1	−2.6	PHLDA3	2.9	HVM51	2.5
			POLK	2.4			MGMT	2.5	CELR3	2.8	IGHG1 *	2.5
							POLE3	2.5	FABP4	2.7		
									MGMT	2.7		
									CA3	2.6		
									LTC4S	2.5		
Kidney medulla	KRT82 *	6.3	MCM3	6.5	S100G	−3.1	MITF	6.9	CCDC8	−3.7	KV2A7	3.0
	RCSD1	−4.0	ECE1	6.4	NCOR1	−3.1	BCHE	−4.4	BCHE	−3.5	BCHE	−2.9
	BCHE	−4.0	BCHE	−3.4	RCSD1	−2.9	CCDC8	−4.4	PHLDA3	3.4	CCDC8	−2.8
	KRT31 *	3.9	BMPR2	−3.3	PLIN1	−2.9	POLR1A	−3.4	KAP	−3.2	LCN2 *	**2.8**
	BMPR2	−3.9	TTC7B	3.3	BMPR2	−2.8	PHLDA3	3.3	POLR1A	−3.2	CHIL3 *	**2.7**
	NCOR1	−3.3	HP	2.9	BCHE	−2.7	POLE3	3.2	KRT16	−2.9	POLR1A	−2.7
	MCM3	3.0	RCSD1	−2.7	YIPF1	−2.7	KAP	−2.9	POLE3	2.8	GCAB	2.6
	**KRT85**	2.8	SAA1	2.6	SAA1	2.6	KLHL8	−2.8	OXR1 C7	−2.7	**TGFB1I1**	2.5
	EHBP1	−2.8	A1BG	−2.5	CDC26	−2.4	KRT16	−2.6	KLHL8	−2.6	KRT71	−2.5
	KT33A	2.7	PHLDA3	2.3	TMCO3	−2.4	MYH1	−2.5	MCM3	2.6	ALDH1A3	−2.4
	A1BG	−2.7	PLIN1	−2.3	FAU	2.4			FAM107B	−2.2	HVM54	2.3
	FRM4B	−2.4	NCOR1	−2.3	SAA2	2.3			SMAGP	−2.2		
	MAVS	−2.4	MAVS	−2.1	ARHGAP23 *	−2.3			MYO9B	−2.2		
	KAP	−2.3			AT.Z.P.5PF	−2.2			COL7A1	−2.2		
	PHLDA3	2.3			HSD17B2	−2.2						
Bone marrow	SFN	−5.3	SFN	−6.5	SFN	−3.9	BCHE	−6.7	BCHE	−5.9	MYH3	6.5
	ABHD5	−3.2	TNNT2	4.7	RSRC2	3.1	NEK1	4.7	MYH3	3.9	ZNF787	−5.4
	HELZ	−3.0	ANKRD2	4.4	ABHD5	−3.1	PIP5K1A	4.1	NEK1	3.5	BCHE	−4.4
	GALNT7	−2.6	TNNI1	3.5	ZNF280D	−2.8	TNNT2	−3.3	PIP5K1A	3.1	MYLPF	3.9
	RSRC2	2.4	ABHD5	−3.5	HELZ	−2.7	MYH7B	−3.2	MYLPF	3.1	MYH4	3.9
	A1BG	−2.2	MYL2	3.4	SMAP	2.5	MYH7	−2.8	TNNT3	3.0	ACTN3	3.8
	XRCC4	−2.2	MYL3	3.4	SAA1	2.4	MYL2	−2.8	CCDC167	−2.9	TNNT3	3.7
			**MYH7**	3.3	KRT71	2.3	KAT5	2.7	ACTN3	2.9	MFF	−3.6
			CRYAB *	3.2	SATB2	2.3	SERPINA1E	−2.6	MYL1	2.8	ZNF318	3.6
			**HSPB6**	3.2	TNNI2	−2.3			TNNC2	2.7	AT.Z.P.2A1	3.5
			CPT1B	3.2	GALNT7	−2.2			ZNF787	−2.7	TMPO	−3.5
			HELZ	−2.9	PLEK.H.A	2.2			AT.Z.P.2A1	2.7	MYL1	3.4
			CKMT2	2.9	RYR2	−2.2			DGCR6	2.7	SARNP	−3.3
			RSRC2	2.9					KAT5	2.6	ACTA1	3.3
			**ACTN2**	2.7					TNNI2	2.6	TNNI2	3.2
			FABP3	2.7					MYH1	2.6	TNNC2	3.0
									MYH4	2.5	RSRC2	−3.0
									ACTA1	2.4	HABP4	−3.0
									**KRT6A**	2.4	GCAB	2.9
									CKS2	−2.4	CDCA2	−2.8
									MAD2L1BP	−2.3	IGHG1 *	2.8
									AMPD1	2.2	MYBPC2	2.8
									TMEM9	−2.2	NEB.L.	2.6
											PTMS *	−2.5
											TTN	2.5
											POLR2M	−2.5
											SERPINA1E	−2.5
											MYOM1	2.4
											KRI1	−2.4
											MAD2L1BP	−2.4
											AMPD1	2.4
											CKS2	−2.4
											SNCA SYN	−2.4

**Table 3:** Affected canonical pathways identified by IPA using protein expression data from mouse bone marrow and kidney cortex and medulla after treatment with ^177^Lu-octreotate (^177^Lu), ^177^Lu-octreotate with A1M (^177^Lu + A1M), or with A1M alone. The z-score predicts the activation state, i.e., z ≤ −2.0 indicates inhibition and z ≥ 2.0 indicates activation.

**Kidney Cortex**					
**Time**	**Group**	**Ingenuity Canonical Pathways**	***p*-Value**	**z-Score**	**Involved Proteins**
24 h	^177^Lu + A1M	Estrogen receptor signaling *	4.37 × 10^−2^	−2.00	ARG2, BAD, NCOR1, RAP2A
7 d	^177^Lu	Aryl hydrocarbon receptor signaling	3.24 × 10^−4^	−2.00	BAX, MCM7, NCOA3, NCOR2, NQO1
**Kidney Medulla**					
**Time**	**Group**	**Ingenuity Canonical Pathways**	***p*** **-Value**	**z-Score**	**Involved Proteins**
24 h	^177^Lu + A1M	3-phosphoinositide biosynthesis	7.94 × 10^−3^	−2.00	PAWR, PIP5K1A, PPP1R1A, PPP1R1B
		Superpathway of inositol phosphate compounds	1.45 × 10^−2^	−2.00	
	A1M	3-phosphoinositide biosynthesis	1.35 × 10^−2^	−2.00	
		Superpathway of inositol phosphate compounds	2.45 × 10^−2^	−2.00	
**Bone Marrow**					
**Time**	**Group**	**Ingenuity Canonical Pathways**	***p*** **-Value**	**z-Score**	**Involved Proteins**
24 h	^177^Lu + A1M	Actin cytoskeleton signaling	7.24 × 10^−10^	2.53	ACTA1, ACTN2, CFL2, MYH1, MYH7, MYH8, MYL2, MYL3, MYL6B, MYLK2, MYLK3, MYLPF, TTN
		ILK signaling	2.19 × 10^−8^	2.11	ACTA1, ACTN2, CFL2, CREBBP, FLNC, MYH1, MYH7, MYH8, MYL2, MYL3, MYL6B
		Hepatic fibrosis signaling pathway	8.13 × 10^−5^	2.53	AXIN1, CREBBP, MYL2, MYL3, MYL6B, MYLK2, MYLK3, MYLPF, TRADD, TTN
		Regulation of actin-based motility by Rho	2.34 × 10^−4^	2.24	ACTA1, MYL2, MYL3, MYL6B, MYLPF
		PAK signaling	3.02 × 10^−4^	2.00	CFL2, MYL2, MYL3, YL6B, MYLPF
		Apelin cardiomyocyte signaling pathway	3.47 × 10^−4^	2.24	AT.Z.P.2A1, MYL2, MYL3, MYL6B, MYLPF
		Signaling by Rho family GT.Z.P.ases	7.08 × 10^−4^	2.45	ACTA1, CFL2, DES, MYL2, MYL3, MYL6B, MYLPF
		Cdc42 signaling	2.34 × 10^−3^	2.24	CFL2, MYL2, MYL3, MYL6B, MYLPF
		Cardiac hypertrophy signaling	3.39 × 10^−3^	2.24	CREBBP, HSPB1, MYL2, MYL3, MYL6B, MYLPF
		Gα12/13 signaling	8.13 × 10^−3^	2.00	MYL2, MYL3, MYL6B, MYLPF
		CXCR4 signaling	1.82 × 10^−2^	2.00	
	A1M	Actin cytoskeleton signaling	5.13 × 10^−3^	−2.00	Actn3, MYH3, MYH4, MYLK3, MYLPF
		ILK signaling	1.62 × 10^−2^	−2.00	Actn3, CREBBP, MYH3, MYH4
7d	^177^Lu	ILK signaling	3.55E × 10^−5^	−2.45	ACTN2, MYH7, MYH7B, MYL2, MYL3, MYL6B
		Phospholipase C signaling	1.45 × 10^−3^	−2.00	ARHGEF18, GNB4, MYL2, MYL3, MYL6B
	^177^Lu + A1M	Calcium signaling	5.01 × 10^−12^	2.24	ACTA1, ACTC1, AT.Z.P.2A1, CACNA2D1, MYH1, MYH3, MYH4, MYH8, MYL1,RYR1, RYR2, TNNC2, TNNI2, TNNT2, TNNT3, Tpm1, Tpm2
		Actin cytoskeleton signaling	4.90 × 10^−6^	3.16	ACTA1, ACTC1, Actn3, MYH1, MYH3, MYH4, MYH8, MYL1, MYLPF, PIP5K1A, TTN
		ILK signaling	6.03 × 10^−5^	2.33	ACTA1, ACTC1, Actn3, FLNC, MYH1, MYH3, MYH4, MYH8, MYL1
		Regulation of actin-based motility by Rho	1.48 × 10^−3^	2.24	ACTA1, ACTC1, MYL1, MYLPF, PIP5K1A
		Signaling by Rho family GT.Z.P.ases	1.74 × 10^−3^	2.12	ACTA1, ACTC1, ARHGEF18, DES, GFAP, MYL1, MYLPF, PIP5K1A
		Integrin signaling *	4.37 × 10^−2^	2.23	ACTA1, ACTC1, Actn3, CAPN7, TTN
	A1M	Calcium signaling	1.62 × 10^−9^	2.00	ACTA1, ACTC1, AT.Z.P.2A1, MYH1, MYH3, MYH4, MYH8, MYL1, MYL2, RYR1, RYR2, TNNC2, TNNI2, TNNT2, TNNT3, Tpm1, Tpm2
		Actin cytoskeleton signaling	5.89 × 10^−6^	2.71	ACTA1, ACTC1, Actn3, MYH1, MYH3, MYH4, MYH8, MYL1, MYL2, MYLK3, MYLPF, PIP5K1A, TTN

* Not significant when considering only molecules and/or relationships in mice.

**Table 4:** Recurrently identified upstream regulators of differentially regulated proteins identified using IPA. Data are given for bone marrow and kidney cortex and medulla after treatment with ^177^Lu-octreotate (^177^Lu), ^177^Lu-octreotate with A1M (^177^Lu + A1M), or with A1M alone. The predicted state of activation was based on the z-score, where z ≤ −2.0 indicates inhibition and z ≥ 2.0 indicates activation.

Upstream Regulator	Tissue	Time	Group	Predicted State	Target Proteins in Dataset
Bvht	Bone marrow	24 h	^177^Lu + A1M	Activated	MYH7, MYL2, MYL3, MYOM1, SMYD1, TNNI1, TNNT2, TTN
		7 d	^177^Lu	Inhibited	MYH7, MYL2, MYL3, TNNI1, TNNT2
DNMT3B	Bone marrow	24 h	A1M	Activated	CASQ1, RYR2, TNNT2, TNNT3
		7 d	^177^Lu	Activated	MYH7, MYH7B, MYL2, MYL3, TNNI1, TNNT2
ETV6-RUNX1	Cortex	24 h	^177^Lu	Activated	CORO1A, GBP2, PSMB9, PT.Z.P.RC
	Medulla	24 h	^177^Lu	Activated	CORO1A, C.Y.BB, GBP2, ITGB2, MGMT, PSMB9, PT.Z.P.RC, STMN1
Ifnar	Cortex	24 h	^177^Lu	Inhibited	GBP2, IFIT1B, PSMB8, PSMB9, TAPBP, VCAM1
			A1M	Inhibited	GBP2, PSMB8, PSMB9, TAP1, TAPBP
IFNG	Cortex	24 h	^177^Lu + A1M	Inhibited	ACE, AIF1, ARG2, BBC3, C1QB, GBP2, HLA-DQB1, Iigp1, PSMB10, PSMB8, PSMB9, Tgtp1/Tgtp2
			A1M	Inhibited	ACE, ARG2, GBP2, HLA-DQB1, Iigp1, PSMB8, PSMB9, SLC2A4, TAP1, TAPBP, Tgtp1/Tgtp2
	Medulla	24 h	^177^Lu	Inhibited	AIF1, ALDH1A3, CD74, C.Y.BB, ECE1, GBP2, HLA-DQA1, HLA-DQB1, PARVG, PPP1R1B, PSMB9, SDC4, SMAGP, Tgtp1/Tgtp2
IL10RA	Cortex	24 h	^177^Lu + A1M	Activated	ARG2, GBP2, Iigp1, LUM, MEP1A, PSMB8, PSMB9, Tgtp1/Tgtp2
		7 d	^177^Lu	Activated	CLIC6, IFI16, LTC4S, Tgtp1/Tgtp2
KDM5A	Bone marrow	24 h	^177^Lu + A1M	Inhibited	ACTN2, FXYD1, MYH7, MYH8, MYL6B, PGAM2, TNNC2, TNNT2, Tpm2, TRIM72
			A1M	Activated	Actn3, FXYD1, MYH4, TNNC2, TNNI2, TNNT2
		7 d	^177^Lu	Activated	ACTN2, MYH7, MYL6B, TNNT2
			^177^Lu + A1M	Inhibited	ACTC1, Actn3, MFN2, MYH4, MYH8, MYL1, PGAM2, RYR1, TNNC2, TNNI2, TNNT2, Tpm1, Tpm2, TRIM72
			A1M	Inhibited	ACTC1, Actn3, MFN2, MYH4, MYH8, MYL1, PGAM2, RYR1, TNNC2, TNNI2, TNNT2, Tpm1, Tpm2, TRIM72
LHX1	Cortex	24 h	^177^Lu + A1M	Inhibited	AADAT, Kap, MEP1A, MEP1B, SLC22A24
			A1M	Inhibited	AADAT, Kap, MEP1A, MEP1B, SLC22A24
mir-21	Cortex	24 h	A1M	Activated	GBP2, Iigp1, TAP1, Tgtp1/Tgtp2
		7 d	^177^Lu + A1M	Activated	AIF1, COL1A1, COL3A1, IGHM, Tgtp1/Tgtp2
	Medulla	24 h	^177^Lu	Activated	AIF1, BMPR2, GBP2, Tgtp1/Tgtp2
MRTFA	Cortex	7 d	A1M	Inhibited	CMA1, LCN2, LTF, Ngp, S100A9
	Medulla	7 d	A1M	Inhibited	CAMP, LCN2, Ngp, S100A9
MRTFB	Cortex	7 d	^177^Lu + A1M	Inhibited	CMA1, LCN2, LTF, Ngp, S100A9
	Medulla	7 d	A1M	Inhibited	CAMP, LCN2, Ngp, S100A9
MYOCD	Bone marrow	24 h	^177^Lu + A1M	Activated	ACTA1, ACTN2, DES, MYH7, MYL2, TNNI1, TNNT2, TTN
		7 d	^177^Lu	Inhibited	ACTN2, MYH7, MYL2, TNNI1, TNNT2
MYOD1	Bone marrow	24 h	^177^Lu + A1M	Activated	ACTA1, ANKRD2, AT.Z.P.2A1, CKM, DES, MYLPF, TNNC2, TNNT2
			A1M	Inhibited	ANKRD2, MYH3, MYH4, MYLPF, TNNC2, TNNI2, TNNT2, TNNT3
		7 d	^177^Lu + A1M	Activated	ACTA1, AT.Z.P.2A1, DES, DM.D., ENO3, INPP5K, MYH3, MYH4, MYL1, MYLPF, TNNC2, TNNI2, TNNT2, TNNT3
			A1M	Activated	ACTA1, AT.Z.P.2A1, DES, ENO3, MYH3, MYH4, MYL1, MYLPF, TNNC2, TNNI2, TNNT2, TNNT3
NOS2	Cortex	7 d	^177^Lu + A1M	Inhibited	BAX, FABP4, FASN, KRT13, MB, Tgtp1/Tgtp2
	Bone marrow	24 h	^177^Lu + A1M	Inhibited	ACTA1, COX6A2, COX7A1, MB, MYH7, MYL2, MYL3, TNNT2
		7d	A1M	Inhibited	ACTA1, ACTC1, CD3E, COX6A2, IGHG1, KRT13, MB, MYL2, TNNT2, TNNT3
NRAS	Cortex	24 h	A1M	Activated	GBP2, Iigp1, PSMB8, TAP1, Tgtp1/Tgtp2
	Medulla	7 d	^177^Lu	Inhibited	BAX, EPHX1, KCTD12, PHLDA3
RB1	Bone marrow	24 h	^177^Lu + A1M	Activated	ACTN2, CKM, COL5A1, FXYD1, MECR, MYH7, MYH8, MYL6B, PGAM2, TNNC2, TNNT2, Tpm2, TRIM72
		7 d	^177^Lu + A1M	Activated	ACTC1, Actn3, BAK1, BCL2L11, Esrra, Krt10, KRT5, LOXL2, MFN2, MYH4, MYH8, MYL1, PGAM2, RYR1, TNNC2, TNNI2, TNNT2, Tpm1, Tpm2, TRIM72, TUBG1, ZNF638
			A1M	Activated	ACTC1, Actn3, BAK1, Krt10, LOXL2, MFN2, MYH4, MYH8, MYL1, PGAM2, RYR1, SAFB, TNNC2, TNNI2, TNNT2, Tpm1, Tpm2, TRIM72, ZNF638
SIRT1	Cortex	24 h	^177^Lu	Activated	BBC3, CORO1A, HLA-DQB1, IFIT1B, Iigp1, PSMB9, Tgtp1/Tgtp2
			^177^Lu + A1M	Activated	BBC3, CORO1A, HLA-DQB1, HMGCR, IFIT1B, Iigp1, PSMB9, Tgtp1/Tgtp2
			A1M	Activated	HLA-DQB1, HMGCR, Iigp1, PSMB9, TAP1, Tgtp1/Tgtp2
SMTNL1	Bone marrow	24 h	^177^Lu + A1M	Inhibited	ACTA1, FLNC, MYOM1, TNNC2, Tpm2
			A1M	Activated	MYH4, TNNC2, TNNI2, TNNT3
		7 d	^177^Lu + A1M	Inhibited	ACTA1, FLNC, MYH4, MYL1, MYOM1, PYGM, TNNC2, TNNI2, TNNT3, Tpm1, Tpm2
			A1M	Inhibited	ACTA1, FLNC, MYH4, MYL1, MYOM1, PYGM, TNNC2, TNNI2, TNNT3, Tpm1, Tpm2
SRF	Bone marrow	24 h	^177^Lu + A1M	Activated	ACTA1, CKM, DES, FHL1, LDB3, MYH1, MYH7, MYL3, MYOM1, Nebl, Tpm2, TTN
		7 d	^177^Lu + A1M	Activated	ACTA1, ACTC1, BCL2L11, DES, DM.D., LDB3, MYH1, MYH4, MYL1, MYOM1, Nebl, Tpm1, Tpm2, TTN, TUBB4B
			A1M	Activated	ACTA1, ACTC1, AKAP12, DES, Igkv1-117, LDB3, MYH1, MYH4, MYL1, MYOM1, Nebl, Tpm1, Tpm2, TTN, TUBB4B
STAT1	Cortex	24 h	^177^Lu	Inhibited	CEACAM1, GBP2, IFIT1B, Iigp1, PSMB10, PSMB8, PSMB9, Tgtp1/Tgtp2
			^177^Lu + A1M	Inhibited	BAD, Cyp2d9 (includes others), GBP2, IFIT1B, Iigp1, PSMB10, PSMB8, PSMB9, Tgtp1/Tgtp2
			A1M	Inhibited	BAD, Cyp2d9 (includes others), GBP2, Iigp1, PSMB8, PSMB9, TAP1, Tgtp1/Tgtp2
	Medulla	24 h	^177^Lu	Inhibited	ALDH1A3, BAD, CAND2, GBP2, HLA-DQA1, PSMB9, SMAGP, Tgtp1/Tgtp2
TRIM24	Cortex	24 h	^177^Lu	Activated	GBP2, IFIT1B, Iigp1, PSMB10, PSMB8, PSMB9, Tgtp1/Tgtp2
			^177^Lu + A1M	Activated	GBP2, IFIT1B, Iigp1, PSMB10, PSMB8, PSMB9, Tgtp1/Tgtp2
			A1M	Activated	GBP2, Iigp1, PSMB8, PSMB9, TAP1, Tgtp1/Tgtp2
	Medulla	24 h	^177^Lu	Activated	GBP2, MGMT, PSMB9, Tgtp1/Tgtp2

**Table 5:** In silico toxicity functions related to nephrotoxicity identified by IPA using protein expression data. Data are given for kidney cortex and medulla after treatment with ^177^Lu-octreotate (^177^Lu), ^177^Lu-octreotate with A1M (^177^Lu + A1M) or with A1M alone. Bias corrected z-score predicts the activation state, i.e., z ≤ −2.0 indicates inhibition and z ≥ 2.0 indicates activation.

**Kidney Cortex**						
**Time**	**Treatment**	**Category**	**Function**	***p*-Value**	**z-Score**	**Target Proteins in Dataset**
7 d	^177^Lu + A1M	Renal inflammation, renal nephritis	Nephritis	1.73 × 10^−2^	−1.88	FABP1, HLA-DQB1, DCN, IGHM, BAX, SIRT1, Uox
**Kidney Medulla**						
**Time**	**Treatment**	**Category**	**Function**	***p*** **-Value**	**z-Score**	**Target Proteins in Dataset**
24 h	^177^Lu	Glomerular injury	Glomerulosclerosis	3.45 × 10^−3^	−1.19 *	Kap, CDKN1B, REN, HMOX1, STMN1
24 h	^177^Lu	Renal necrosis/ cell death	Cell death	3.17 × 10^−2^	−0.81	MAVS, CDKN1B, SOD1, C.Y.BB, BAD, STMN1
7 d	^177^Lu + A1M	Renal necrosis/ cell death **	Cell viability	8.66 × 10^−4^	1.45	CAV1, BAX, ABCC10, MAPT
7 d	A1M	Renal necrosis/ cell death **	Cell death	2.69 × 10^−2^	0.14	PTGDS, TGFB1I1, SOD1, CALB1, LCN2

* No bias correction of the z-score was made; ** not found when considering molecules and/or relationships in mice only.

## Data Availability

The data presented in this study are openly available in the Proteomic identifications database (Project accession: PXD029937).

## Supplementary Materials

The supporting information can be downloaded at: https://www.mdpi.com/article/10.3390/ijms25137480/s1.

## References

[1] Olsson M.G., Olofsson T., Tapper H., Akerstrom B. (2008). The lipocalin alpha1-microglobulin protects erythroid K562 cells against oxidative damage induced by heme and reactive oxygen species. Free Radic. Res..

[2] Garske-Román U., Sandström M., Fröss Baron K., Lundin L., Hellman P., Welin S., Johansson S., Khan T., Lundqvist H., Eriksson B. (2018). Prospective observational study of (177)Lu-DOTA-octreotate therapy in 200 patients with advanced metastasized neuroendocrine tumours (NETs): Feasibility and impact of a dosimetry-guided study protocol on outcome and toxicity. Eur. J. Nucl. Med. Mol. Imaging.

[3] Larsson M., Bernhardt P., Svensson J.B., Wängberg B., Ahlman H., Forssell-Aronsson E. (2012). Estimation of absorbed dose to the kidneys in patients after treatment with 177Lu-octreotate: Comparison between methods based on planar scintigraphy. EJNMMI Res..

[4] Twardella D., Chang-Claude J. (2002). Studies on radiosensitivity from an epidemiological point of view—Overview of methods and results. Radiother. Oncol. J. Eur. Soc. Ther. Radiol. Oncol..

[5] Geenen L., Nonnekens J., Konijnenberg M., Baatout S., De Jong M., Aerts A. (2021). Overcoming nephrotoxicity in peptide receptor radionuclide therapy using [(177)Lu]Lu-DOTA-TATE for the treatment of neuroendocrine tumours. Nucl. Med. Biol..

[6] Rolleman E.J., Valkema R., de Jong M., Kooij P.P., Krenning E.P. (2003). Safe and effective inhibition of renal uptake of radiolabelled octreotide by a combination of lysine and arginine. Eur. J. Nucl. Med. Mol. Imaging.

[7] Rolleman E.J., Bernard B.F., Breeman W.A., Forrer F., de Blois E., Hoppin J., Gotthardt M., Boerman O.C., Krenning E.P., de Jong M. (2008). Molecular imaging of reduced renal uptake of radiolabelled [DOTA0,Tyr3]octreotate by the combination of lysine and Gelofusine in rats. Nuklearmedizin. Nucl. Med..

[8] Okunieff P., Swarts S., Keng P., Sun W., Wang W., Kim J., Yang S., Zhang H., Liu C., Williams J.P. (2008). Antioxidants reduce consequences of radiation exposure. Adv. Exp. Med. Biol..

[9] Kristiansson A., Örbom A., Vilhelmsson Timmermand O., Ahlstedt J., Strand S.E., Åkerström B. (2021). Kidney protection with the radical scavenger α1-microglobulin (A1M) during peptide receptor radionuclide and radioligand therapy. Antioxidants.

[10] Åkerstrom B., Gram M. (2014). A1M, an extravascular tissue cleaning and housekeeping protein. Free Radic. Biol. Med..

[11] Ahlstedt J., Tran T.A., Strand F., Holmqvist B., Strand S.E., Gram M., Akerstrom B. (2015). Biodistribution and pharmacokinetics of recombinant alpha1-microglobulin and its potential use in radioprotection of kidneys. Am. J. Nucl. Med. Mol. Imaging.

[12] Alattar A.G., Kristiansson A., Karlsson H., Vallius S., Ahlstedt J., Forssell-Aronsson E., Åkerström B., Strand S.E., Flygare J., Gram M. (2023). Recombinant α1-microglobulin (rA1M) protects against hematopoietic and renal toxicity, alone and in combination with amino acids, in a 177Lu-DOTATATE mouse radiation mode. Biomolecules.

[13] Kristiansson A., Ahlstedt J., Holmqvist B., Brinte A., Tran T.A., Forssell-Aronsson E., Strand S.E., Gram M., Åkerström B. (2019). Protection of Kidney Function with Human Antioxidation Protein α(1)-Microglobulin in a Mouse (177)Lu-DOTATATE Radiation Therapy Model. Antioxid Redox Signal.

[14] Andersson C.K., Shubbar E., Schüler E., Åkerström B., Gram M., Forssell-Aronsson E.B. (2019). Recombinant α(1)-Microglobulin Is a Potential Kidney Protector in (177)Lu-Octreotate Treatment of Neuroendocrine Tumors. J. Nucl. Med. Off. Publ. Soc. Nucl. Med..

[15] Verburg F.A., Nonnekens J., Konijnenberg M.W., de Jong M. (2021). To go where no one has gone before: The necessity of radiobiology studies for exploration beyond the limits of the “Holy Gray” in radionuclide therapy. Eur. J. Nucl. Med. Mol. Imaging.

[16] Aerts A., Eberlein U., Holm S., Hustinx R., Konijnenberg M., Strigari L., van Leeuwen F.W.B., Glatting G., Lassmann M. (2021). EANM position paper on the role of radiobiology in nuclear medicine. Eur. J. Nucl. Med. Mol. Imaging.

[17] Andreassen C.N., Schack L.M., Laursen L.V., Alsner J. (2016). Radiogenomics—Current status, challenges and future directions. Cancer Lett..

[18] Leszczynski D. (2014). Radiation proteomics: A brief overview. Proteomics.

[19] Schüler E., Rudqvist N., Parris T.Z., Langen B., Helou K., Forssell-Aronsson E. (2014). Transcriptional response of kidney tissue after 177Lu-octreotate administration in mice. Nucl. Med. Biol..

[20] Schüler E., Rudqvist N., Parris T.Z., Langen B., Spetz J., Helou K., Forssell-Aronsson E. (2014). Time- and dose rate-related effects of internal (177)Lu exposure on gene expression in mouse kidney tissue. Nucl. Med. Biol..

[21] Schüler E., Larsson M., Parris T.Z., Johansson M.E., Helou K., Forssell-Aronsson E. (2015). Potential Biomarkers for Radiation-Induced Renal Toxicity following 177Lu-Octreotate Administration in Mice. PLoS ONE.

[22] Schüler E., Parris T.Z., Helou K., Forssell-Aronsson E. (2014). Distinct microRNA expression profiles in mouse renal cortical tissue after 177Lu-octreotate administration. PLoS ONE.

[23] Andersson M. (2019). Apoptotic Effects in Renal Cortex after Treatment with 177Lu-Octreotate.

[24] Merle N.S., Grunenwald A., Figueres M.-L., Chauvet S., Daugan M., Knockaert S., Robe-Rybkine T., Noe R., May O., Frimat M. (2018). Characterization of Renal Injury and Inflammation in an Experimental Model of Intravascular Hemolysis. Front. Immunol..

[25] Djudjaj S., Papasotiriou M., Bülow R.D., Wagnerova A., Lindenmeyer M.T., Cohen C.D., Strnad P., Goumenos D.S., Floege J., Boor P. (2016). Keratins are novel markers of renal epithelial cell injury. Kidney Int..

[26] Bhandari S., Watson N., Long E., Sharpe S., Zhong W., Xu S.Z., Atkin S.L. (2008). Expression of somatostatin and somatostatin receptor subtypes 1-5 in human normal and diseased kidney. J. Histochem. Cytochem. Off. J. Histochem. Soc..

[27] Vaidya V.S., Ferguson M.A., Bonventre J.V. (2008). Biomarkers of acute kidney injury. Annu. Rev. Pharmacol. Toxicol..

[28] Li S., Lu X., Feng J.B., Tian M., Liu Q.J. (2017). Identification and Validation of Candidate Radiation-responsive Genes for Human Biodosimetr. Biomed. Environ. Sci. BES.

[29] Kultova G., Tichy A., Rehulkova H., Myslivcova-Fucikova A. (2020). The hunt for radiation biomarkers: Current situation. Int. J. Radiat. Biol..

[30] Marchetti F., Coleman M.A., Jones I.M., Wyrobek A.J. (2006). Candidate protein biodosimeters of human exposure to ionizing radiation. Int. J. Radiat. Biol..

[31] Chipuk J.E., Moldoveanu T., Llambi F., Parsons M.J., Green D.R. (2010). The BCL-2 family reunion. Mol. Cell.

[32] Huang J., Qi Z., Chen M., Xiao T., Guan J., Zhou M., Wang Q., Lin Z., Wang Z. (2019). Serum amyloid A1 as a biomarker for radiation dose estimation and lethality prediction in irradiated mouse. Ann. Transl. Med..

[33] Åkerstrom B., Logdberg L., Berggard T., Osmark P., Lindqvist A. (2000). alpha(1)-Microglobulin: A yellow-brown lipocalin. Biochim. Et Biophys. Acta.

[34] Chen C., Lorimore S.A., Evans C.A., Whetton A.D., Wright E.G. (2005). A proteomic analysis of murine bone marrow and its response to ionizing radiation. Proteomics.

[35] Magić Z., Matić-Ivanović S., Savić J., Poznanović G. (1995). Ionizing radiation-induced expression of the genes associated with the acute response to injury in the rat. Radiat. Res..

[36] Bergwik J., Kristiansson A., Allhorn M., Gram M., Åkerström B. (2021). Structure, Functions, and Physiological Roles of the Lipocalin α(1)-Microglobulin (A1M). Front. Physiol..

[37] Andersen C.B.F., Stødkilde K., Sæderup K.L., Kuhlee A., Raunser S., Graversen J.H., Moestrup S.K. (2017). Haptoglobin. Antioxid. Redox Signal..

[38] Kristiansson A., Bergwik J., Alattar A.G., Flygare J., Gram M., Hansson S.R., Olsson M.L., Storry J.R., Allhorn M., Åkerström B. (2021). Human radical scavenger α(1)-microglobulin protects against hemolysis in vitro and α(1)-microglobulin knockout mice exhibit a macrocytic anemia phenotype. Free Radic. Biol. Med..

[39] Wester L., Michaëlsson E., Holmdahl R., Olofsson T., Akerström B. (1998). Receptor for alpha1-microglobulin on T lymphocytes: Inhibition of antigen-induced interleukin-2 production. Scand. J. Immunol..

[40] Fernandez-Luna J.L., Leyva-Cobian F., Mollinedo F. (1988). Identification of the protein HC receptor. FEBS Lett..

[41] Olmos G., López-Ongil S., Ruiz Torres M.P. (2017). Integrin-linked kinase: A new actor in the ageing process?. Exp. Gerontol..

[42] Guan Y., Hao C.M. (2016). SIRT1 and Kidney Function. Kidney Dis..

[43] Najjar I., Fagard R. (2010). STAT1 and pathogens, not a friendly relationship. Biochimie.

[44] Bhayana S., Song F., Jacob J., Fadda P., Denko N.C., Xu-Welliver M., Chakravarti A., Jacob N.K. (2017). Urinary miRNAs as Biomarkers for Noninvasive Evaluation of Radiation-Induced Renal Tubular Injury. Radiat. Res..

[45] Åkerström B., Rosenlöf L., Hägerwall A., Rutardottir S., Ahlstedt J., Johansson M.E., Erlandsson L., Allhorn M., Gram M. (2019). rA1M-035, a Physicochemically Improved Human Recombinant α(1)-Microglobulin, Has Therapeutic Effects in Rhabdomyolysis-Induced Acute Kidney Injury. Antioxid. Redox Signal..

[46] Bolch W.E., Eckerman K.F., Sgouros G., Thomas S.R. (2009). MIRD pamphlet No. 21: A generalized schema for radiopharmaceutical dosimetry—Standardization of nomenclature. J. Nucl. Med. Off. Publ. Soc. Nucl. Med..

[47] Eckerman K., Endo A. (2008). ICRP Publication 107. Nuclear Decay Data for Dosimetric Calculations. Ann ICRP.

[48] Schüler E., Österlund A., Forssell-Aronsson E. (2016). The amount of injected 177Lu-octreotate strongly influences biodistribution and dosimetry in C57BL/6N mice. Acta Oncol..

[49] Svensson J., Mölne J., Forssell-Aronsson E., Konijnenberg M., Bernhardt P. (2012). Nephrotoxicity profiles and threshold dose values for [177Lu]-DOTATATE in nude mice. Nucl. Med. Biol..

[50] Wiśniewski J.R., Zougman A., Nagaraj N., Mann M. (2009). Universal sample preparation method for proteome analysis. Nat. Methods.

