# Supplementary material for: Effects of Recombinant α1-Microglobulin on Early Proteomic Response in Risk Organs after Exposure to 177Lu-Octreotate
Source: Int J Mol Sci. 2024 Jul 8;25(13):7480. doi: 10.3390/ijms25137480 (PMC11242497; doi:10.3390/ijms25137480)
Supplement: Supplementary file 1 [file ijms-25-07480-s001.zip › ijms-3048391-supplementary.pdf]

Supplemental data

Supplemental Table S1. Protein regulation in mouse kidney cortex at 24 hours and 7 days after injection of ^{177}Lu-octreotate (^{177}Lu), ^{177}Lu-octreotate with A1M (^{177}Lu + A1M) or A1M alone. Data is given for differentially regulated proteins (DRP) with statistically significant difference between any of the groups. FC ≥ 1.5 means upregulation (blue) and FC ≤ -1.5 means down regulation (orange) compared with control. * statistically significant difference found in the pairwise comparison with Welch´s test that followed the 1-way ANOVA.

Kidney cortex

24 h

	Protein FC			Statistical significance		
**Gene encoding the DRP**	**^{177}Lu**	**^{177}Lu + A1M**	**A1M**	**^{177}Lu *vs* ^{177}Lu + A1M**	**^{177}Lu *vs* A1M**	**^{177}Lu + A1M *vs* A1M**
**Aadat Kat2**	1.2	-1.6	-1.8	*	*	
**Ace Dcp1**	1.2	-1.5	-1.8	*	*	
**Acox2**	1.2	-1.4	-1.5	*	*	
**Aif1 Iba1**	-1.8	-1.5	-1.1		*	
**Akr1c18**	1.2	-1.8	-2.0		*	
**Arg2**	1.5	-1.6	-1.9	*	*	
**Baz1a Cbp146**	-1.0	-1.2	-1.5		*	
**Bbc3 Puma**	2.2	2.0	-1.4		*	*
**Cd99l2 Mic2l1**	-1.9	-1.3	-1.2	*	*	
**Ceacam2 Bgp2**	1.9	-1.1	-1.4	*	*	
**Cep120 Ccdc100**	-2.0	-2.1	-1.7		*	
**Ces1f CesML1**	1.1	-1.3	-1.5	*	*	
**Ces2e Ces5**	2.0	2.1	1.4		*	
**Chd1 Chd-1**	1.6	1.2	1.0	*	*	
**Cmc2**	1.1	-1.6	-2.1	*	*	
**Coro1a Coro1**	-1.8	-1.6	-1.1	*	*	*
**Cryab Crya2**	2.2	1.1	-1.1	*	*	
**Cyp2a4 Cyp2a-4**	1.1	-2.5	-2.8	*	*	
**Cyp51a1 Cyp51**	-1.1	-1.5	-1.3	*	*	
**Ephx1**	1.8	1.6	1.0	*	*	*
**Fem1c Kiaa1785**	1.6	1.4	1.4	*	*	
**H2-Ab1 H2-iabeta**	-2.7	-3.0	-2.1		*	*
**Hp**	1.2	3.5	1.4	*		*
**Ifit1 Garg16 Ifi56 Isg56**	-1.6	-1.6	-1.1		*	
**Kap**	1.2	-1.7	-2.2	*	*	
**Krt71 K6irs1 Kb34 Krt2-6g Krt6g**	1.4	2.6	1.7	*		
**Lamtor5 Hbxip Xip**	-1.7	-1.3	1.1	*	*	
**Lsp1 Pp52 S37 Wp34**	-3.0	-1.9	-1.2	*	*	*

7 d

	Protein FC			Statistical significance		
**Gene encoding the DRP**	**^{177}Lu**	**^{177}Lu + A1M**	**A1M**	**^{177}Lu *vs* ^{177}Lu + A1M**	**^{177}Lu *vs* A1M**	**^{177}Lu + A1M *vs* A1M**
Acaca Acac Gm738	-1.1	1.9	1.1			
Actr8 Arp8	-1.6	-1.2	-1.8	*		*
Aida	1.4	1.2	1.5			*
Aif1 Iba1	-2.6	-1.9	1.3			*
Aldh1a7 Aldh-pb	1.5	1.8	1.3			*
Arhgap45 Hmha1	-1.5	-1.4	1.1			*
Arhgdib Gdid4	1.3	1.2	1.6			*
Arpp19	-1.4	1.3	-1.7	*		*
Axin1 Axin Fu	-1.8	-1.3	-1.4	*		
Bax	1.6	1.6	-1.0		*	*
Ca3 Car3	-1.2	2.6	-1.0			*
Cav1 Cav	-1.1	1.8	-1.1	*		*
Cavin1 Ptrf	1.1	1.6	1.1			*
Cbr3	1.6	1.5	1.1		*	*
Celsr3	1.3	2.8	1.7			*
Ces2e Ces5	1.9	2.0	-1.0		*	*
Clcnka Clcnk1	1.2	1.1	1.9			*
Cldn10 Cldn10a	-1.3	-1.5	1.0			*
Clic6	1.6	-1.6	1.0	*	*	*
Coro1a Coro1	-2.0	-1.9	1.3			*
Cpne4	-2.0	-1.8	-1.2		*	*
Dennd5b	1.0	1.7	1.3	*		*
Dglucy	1.7	1.7	1.0		*	*
Dixdc1 Ccd1 Kiaa1735	1.1	1.6	-1.0	*		*
Edrf1	-1.3	2.9	1.2	*		*
Ehbp1 Kiaa0903	1.1	1.7	-1.0			*
Eif4ebp2	-1.3	2.2	-1.8			*
Ephx1	3.2	3.1	1.1		*	*

**Map3k20 Mltk Zak**	1.6	1.6	1.1		*	*
**Mep1a**	1.2	-1.5	-1.9	*	*	
**Mep1b Mep-1b**	1.3	-1.8	-2.5	*	*	
**Morc3 Nxp2 Zcwcc3**	1.0	-1.3	-1.5	*	*	
**Nisch Kiaa0975**	-1.6	-1.1	-1.0	*	*	
**Phlda3 Tih1**	2.2	2.3	1.0		*	*
**Polk Dinb1**	1.8	2.4	1.4	*	*	
**Saa1**	1.0	2.4	2.7	*	*	
**Saa2**	1.9	3.5	4.7		*	
**Slc22a13 Orctl3**	1.1	-1.7	-2.2	*	*	
**Slc22a19 Oat5 Slc22a9**	1.2	-1.8	-2.5	*	*	
**Slco1a6 Oatp5 Slc21a13**	1.0	-1.3	-1.9	*	*	
**Usp2 Ubp41 MNCb-0190**	-1.0	-1.7	-1.9	*	*	
**Yipf1**	-1.1	-1.6	-2.0	*	*	

Ercc5 Ercc-5 Xpg	-1.1	1.3	-1.6	*		*
Fmnl3 Frl2 Kiaa2014	1.3	1.2	1.5			*
Gbp2	-1.1	-1.1	1.6			*
Gorasp1	-1.1	1.6	-1.2			*
H2-Ab1 H2-iabeta	-1.6	-1.5	1.3			*
Homer3	1.2	2.2	1.2			*
Hpd	-1.1	-1.6	-1.1	*		*
Igf2bp1 Vickz1	1.1	1.6	-1.1	*		*
Inmt Temt	-1.2	-1.5	1.1		*	*
Itm2b	1.5	1.6	1.2		*	*
Kctd12 Pfet1	1.9	1.8	1.1		*	*
Klk1 Klk-6 Klk6	-1.4	-1.5	-1.2			*
Ltc4s	2.3	2.5	-1.0		*	*
Marcksl1 Mlp Mrp	-1.0	1.7	-1.4	*		*
Mcm2 Bm28 Cdcl1 Kiaa0030 Mcmd2	-2.2	-2.3	-1.0		*	*
Mcm3 Mcmd Mcmd3	-2.4	-2.4	-1.0		*	*
Mcm7 Cdc47 Mcmd7	-1.5	-1.5	-1.0		*	*
Mgmt	2.5	2.7	-1.1		*	*
Nckap1l Hem1	-1.7	-1.4	1.3	*		*
Ncoa3 Aib1 Pcip Rac3 Tram1	-1.6	1.1	-1.4	*		*
Ncor2 Smrt	-1.6	-1.2	-1.5	*		*
Nqo1 Dia4 Nmo1 Nmor1	1.5	1.3	1.1			*
Ociad1 Asrij	-1.0	1.4	-1.5	*		*
Palm3	1.5	1.5	1.1		*	*
Pcbp2 Cbp Hnrnpx Hnrpx	-1.7	-1.4	-1.7	*		*
Pcnp	-1.7	1.2	-1.6			*
Phlda3 Tih1	2.5	2.9	1.0		*	*
Plcd4 Plcd	2.8	2.9	1.2		*	*
Polk Dinb1	2.8	6.5	2.6			*
Psmb9	-1.3	-1.1	1.6			*
Rgs10	-2.0	-1.9	1.2		*	*
Rnasek D11Bwg0434e	1.0	-1.5	-1.1			*
Rpl36a Rpl44	-1.1	-2.3	-1.1			*
Rsrp1 D4Wsu53e MNCb-0169	-1.6	1.6	-1.6	*		*
Sirt1 Sir2l1	-1.1	1.7	-1.4			*
Stx18	1.6	1.4	1.5			*

	Tbc1d4<br>As160<br>Kiaa0603	-2.3	-2.1	-1.8		*	*
	Tdrp	-1.4	1.2	-1.7	*		*
	Tgtp1<br>Ifggb5 Irgb6<br>Mg21	-1.6	-1.7	1.6			*
	Thyn1<br>Thy28	1.5	1.8	-1.2		*	*
	Tmem259<br>ORF61	-1.5	1.2	-1.7			*
	Tmem43	1.5	1.7	1.1		*	*
	Tp53bp1<br>Trp53bp1	-1.5	-1.2	-1.1			
	Trim36	-1.9	-1.4	-1.1		*	
	Ugt1a6<br>Ugt1<br>Ugt1a6a<br>Ugt1a7	1.5	1.5	1.1		*	*
	Urgcp Urg4	-1.8	-1.7	-1.5		*	*
	Wdr24	1.4	1.9	1.2			*

Supplemental Table S2. Protein regulation in mouse kidney medulla at 24 hours and 7 days after injection of ^{177}Lu-octreotate (^{177}Lu), ^{177}Lu-octreotate with A1M (^{177}Lu + A1M) or with A1M alone. Data is given for differentially regulated proteins (DRP) with statistically significant difference between any of the groups. FC ≥ 1.5 means upregulation (blue) and FC ≤ -1.5 means down regulation (orange) compared with control. * statistically significant difference found in the pairwise comparison with Welch´s test that followed the 1-way ANOVA.

Kidney medulla

24 h

	Protein FC			Statistical significance		
**Gene encoding the DRP**	**^{177}Lu**	**^{177}Lu + A1M**	**A1M**	**^{177}Lu *vs* ^{177}Lu+A1M**	**^{177}Lu *vs* A1M**	**^{177}Lu+A1M *vs* A1M**
**Itgb2**	-1.9	-1.3	-1.1	*	*	*
**Mgmt**	1.6	1.7	1.0		*	*
**Phlda3 Tih1**	2.3	2.3	1.1		*	*
**Rfx5**	-1.2	-1.2	-1.6		*	*

7 d

	Protein FC			Statistical significance		
**Gene encoding the DRP**	**^{177}Lu**	**^{177}Lu +A1M**	**A1M**	**^{177}Lu *vs* ^{177}Lu+A1M**	**^{177}Lu *vs* A1M**	**^{177}Lu+A1M *vs* A1M**
Bax	1.5	1.5	1.1		*	*
Cyp2d9 Cyp2d-9	-1.1	1.1	1.7		*	*
Ephx1	2.2	2.0	-1.0		*	*
Kctd12 Pfet1	1.5	1.5	1.0		*	*
Mgmt	1.8	1.8	-1.1		*	*
N/A (Protein name: C19orf12	-1.0	-1.2	1.5		*	*
Phlda3 Tih1	3.3	3.4	1.0		*	*
Serpinb1a Serpinb1	-1.5	-2.1	-1.1	*	*	*
Sfswap Sfrs8 Srsf8 Swap	1.4	-1.5	-1.1	*	*	*

Supplemental Table S3. Protein regulation in mouse bone marrow at 24 hours and 7 days after injection of ^{177}Lu-octreotate (^{177}Lu), ^{177}Lu-octreotate with A1M (^{177}Lu + A1M) or with A1M alone. Data is given for differentially regulated proteins (DRP) with statistically significant difference between any of the groups. FC ≥ 1.5 means upregulation (blue) and FC ≤ -1.5 means down regulation (orange) compared with control. * statistically significant difference found in the pairwise comparison with Welch´s test that followed the 1-way ANOVA.

Bone marrow

24 h

	Protein FC			Statistical significance		
**Gene encoding the DRP**	**^{177}Lu**	**^{177}Lu +A1M**	**A1M**	**^{177}Lu *vs* ^{177}Lu+ A1M**	**^{177}Lu *vs* A1M**	**^{177}Lu+ A1M *vs* A1M**
**Atp13a3 Gm542**	1.1	1.2	1.5		*	*
**Faf2 Kiaa0887 Ubxd8**	1.3	1.4	1.6	*	*	*
**Snupn Rnut1**	-1.1	-1.4	-1.6	*	*	*

7 d

	Protein FC			Statistical significance		
**Gene encoding the DRP**	**^{177}Lu**	**^{177}Lu +A1M**	**A1M**	**^{177}Lu *vs* ^{177}Lu+ A1M**	**^{177}Lu *vs* A1M**	**^{177}Lu+ A1M *vs* A1M**
Abca1 Abc1	1.1	-1.4	-1.6	*	*	
Api5	-1.4	-1.5	-1.5		*	
Apoa2	-1.2	-1.2	-1.6		*	
Arhgef18 Kiaa0521	-1.8	-2.1	-1.7	*		
Ate1	-1.1	-1.6	-1.4	*	*	
Atp13a3 Gm542	1.0	-2.2	-2.1	*	*	
Bak1 Bak	-1.0	-1.7	-1.5	*	*	
Bcar3 And34	-1.3	-1.5	-1.9		*	
Bcl2l11 Bim	-1.0	-1.6	-1.4	*	*	
Ccdc167	-2.1	-2.9	-2.2	*		
Ccr7 Cmkbr7 Ebi1 Ebi1h	-1.0	-1.6	1.0	*		
Cd3e	-1.0	1.7	1.7	*	*	
Cd79a Iga Mb-1	1.1	1.4	1.6	*	*	
Cd79b Igb	-1.4	-2.2	-1.0	*		
Cers2 Lass2 Trh3	-1.0	-1.6	-1.5	*	*	
Cgref1 Cgr11	1.2	-1.5	-1.9	*	*	
Chchd2	-1.3	-1.8	-1.8	*	*	
Chmp2b	-1.1	-1.0	-1.5		*	
Cks2	-1.2	-2.4	-2.4	*	*	
Clec12a Micl	-1.1	-1.6	-1.4	*	*	
Cnbp Cnbp1 Znf9	-1.6	-1.3	-2.1		*	
Cyp2f2 Cyp2f-2	1.2	1.6	1.0	*		
Dbnl Abp1 Sh3p7	-1.3	-1.6	-1.7	*	*	
Dmtn Epb4.9 Epb49	-1.2	-1.2	-1.6		*	
Dpm3	1.1	-1.6	-1.2	*		
Ebna1bp2 Ebp2	1.0	-1.5	-1.6	*	*	
Eif4h Wbscr1	-1.0	-1.5	-1.6	*	*	
Ercc3 Xpb Xpbc	-1.0	-1.6	-1.3	*	*	
Faf2 Kiaa0887 Ubxd8	-1.3	-1.9	-2.0	*	*	
Fam114a2	-1.1	-1.3	-1.5	*	*	
Fam177a1	-1.2	-1.7	-1.6	*	*	
Fpr2 Fpr-rs2	1.1	-1.5	-1.1	*	*	
Ftl1 Ftl Ftl-1	1.3	1.3	-1.5		*	

Fus	-1.0	-1.5	-1.6	*	*
Ggnbp2 Zfp403 Znf403	1.0	-1.4	-1.5	*	*
Glrx5	1.0	-1.4	-1.8	*	*
Glt8d1	-1.0	-1.4	-1.6	*	*
Gnb4	-1.6	-1.2	1.0	*	*
Gopc	-1.1	-1.4	-1.8		*
Gpn3 Atpbd1c D5Ertd708e	-1.1	-1.6	-1.5	*	*
Gpr84	-1.2	-1.5	-1.6	*	*
Gtpbp3	-1.2	-1.6	-1.8	*	*
Gypa	-1.3	-1.1	-1.8		*
Gzma Ctla-3 Ctla3 Mtsp-1	-1.6	-1.6	-1.0		*
H2-Eb1	-1.6	-1.7	1.1		*
Habp4	-1.2	-1.5	-3.0		*
Hddc2	-1.1	-2.0	-1.8	*	*
Hnrnpa0 Hnrpa0	-1.3	-1.4	-1.8		*
Imp3	1.1	-1.8	-1.5	*	*
Isg20	-1.1	-1.6	-1.5	*	*
Kmt2e Mll5	1.0	-1.1	-1.8		*
Lamtor1	-1.1	-1.5	-1.9	*	*
Loxl2	1.2	1.7	1.6	*	*
Lum Lcn Ldc	1.5	1.1	1.1	*	*
Mcph1	-1.2	-1.3	-2.2		*
Med21 Srb7 Surb7	-1.1	-2.0	-1.8	*	*
Mfn2 Kiaa0214 Marf	-1.2	-1.7	-1.9	*	*
Mknk2 Mnk2 Morf4l1	-1.0	-1.5	-1.5	*	*
Mrg15 Tex189	-1.0	-1.6	-1.5	*	*
Mphosph10	1.0	-1.5	-1.6	*	*
Mpp1	-1.2	-1.2	-1.9		*
Mpp7	-1.2	-1.6	-1.6	*	*
N/A (protein name: Ig delta chain C region membrane-bound form)	-1.5	-2.1	1.1	*	
Nemp1 Kiaa0286 Tmem194 Tmem194a	-1.2	-2.1	-1.9	*	*
Nosip	-1.4	-1.5	-1.9		*
Nudcd2 D11Ertd603e	-1.1	-1.4	-1.6	*	*
Nup35 Mp44 Nup53	-1.0	-1.3	-1.6	*	*
Pbx2	1.0	-1.6	-1.9	*	*
Pcgf6 Mblr Rnf134	1.4	1.7	2.3		*
Pdxdc1	1.1	-1.4	-1.6	*	*
Pin4	-1.0	-1.7	-1.5	*	*
Prorsd1 Prdxdd1	-1.1	-1.6	-1.5	*	*
Ptma	1.1	-1.1	-1.8		*
Ptms	1.1	-1.1	-2.5		*
Rars2 Rarsl	-1.1	-1.4	-1.5	*	*
Rcn2	-1.1	-1.6	-1.8	*	*
Rgs19	1.0	-1.7	-1.4	*	*

Rgs3	-1.2	-1.6	-1.4	*	*
Rnf126	-1.3	-1.2	-1.6		*
Rpp40	-1.1	-1.7	-1.6	*	*
Ryr2	-1.1	1.8	1.7	*	*
Scamp3	-1.3	-1.6	-1.5	*	*
Sdf4 Cab45	-1.0	-1.4	-1.5	*	*
Serpina1e Dom5 Spi1-5	-2.6	-1.4	-2.5	*	
Slc31a2	-1.1	-1.5	-1.6	*	*
Slc38a2 Ata2 Kiaa1382 Sat2 Snat2	1.3	1.6	1.8		*
Srsf5 Hrs Sfrs5	-1.1	-1.6	-1.5	*	*
Syngr2	1.1	1.7	1.6	*	*
Taf11	-1.1	-1.2	-1.6		*
Taf2	-1.1	-1.7	-1.7	*	*
Tbcel Lrrc35	-1.2	-1.5	-1.5	*	*
Tex2 Kiaa1738	-1.3	-1.8	-2.2	*	*
Thg1l	-1.1	-1.8	-2.1	*	*
Tiprl	-1.0	-2.1	-1.7	*	*
Tmem134	-1.1	-1.5	-1.8	*	*
Tmem230	-1.2	-2.2	-2.1	*	*
Tmem38b D4Ertd89e Mg33b	-1.2	-1.4	-1.7		*
Tmem9	-1.2	-2.2	-2.3	*	*
Tmtc3	-1.2	-1.3	-1.6		*
Tomm20	-1.2	-1.4	-2.0		*
Tuba1c Tuba6	-1.2	-2.2	-2.0	*	*
Tubb2a Tubb2	-1.2	-1.8	-2.0	*	*
Tubb4b Tubb2c	-1.1	-1.5	-1.6	*	*
Tubg1 Tubg	-1.1	-1.5	-1.5	*	*
Ube2b Rad6b	-1.1	-1.7	-1.4	*	*
Usp1	-1.1	-1.6	-1.7	*	*
Utp20 Drim	-1.8	-2.0	-1.8		
Vamp3 Syb3	-1.3	-1.5	-2.0		*
Vps25 D11Wsu68e	1.0	-1.6	-1.4	*	*
Washc2 D6Wsu116e Fam21 Kiaa0592	-1.1	-1.4	-1.6	*	*
Yipf4	-1.1	-1.7	-2.0	*	*
Zc3h15 Dfrp1	-1.1	-1.3	-1.6		*
Zfand1	-1.1	1.6	1.8	*	*
Zfp36l2 Brf2 Tis11d	-1.2	-1.2	-1.6		*
Znf24 Hmcns Zfp191 Zfp24	1.1	1.6	1.5	*	*
Znf280d Suhw4 Zfp280d	1.0	2.0	1.5	*	*
Znf638 Np220 Zfml Zfp638	-1.0	-1.7	-1.8	*	*

Supplemental Table S4. Upstream regulators of differentially regulated proteins (|FC| ≥ 1.5) identified by IPA in kidney cortex, kidney medulla and bone marrow from mice injected with ^{177}Lu-octreotate (^{177}Lu), ^{177}Lu-octreotate with A1M (^{177}Lu + A1M) or with A1M alone. Protein data were received from two time-points, 24 hours and 7 days post injection. Z -score predicts activation state, where z ≤ -2.0 indicates inhibition and z ≥ 2.0 indicates activation.

Kidney cortex

Time	Group	Upstream Regulator	Molecule type	p-value	z-score	Target molecules in dataset
**24 h**	^{177}Lu	TRIM24	transcription regulator	5.66×10^{-8}	2.63*	GBP2, IFIT1B, Iigp1, PSMB10, PSMB8, PSMB9, Tgtp1/Tgtp2
		Ifnar	group	4.40×10^{-7}	-2.41*	GBP2, IFIT1B, PSMB8, PSMB9, TAPBP, VCAM1
		STAT1**	transcription regulator	1.41×10^{-6}	-2.00*	CEACAM1, GBP2, IFIT1B, Iigp1, PSMB10, PSMB8, PSMB9, Tgtp1/Tgtp2
		SIRT1****	transcription regulator	7.85×10^{-5}	1.98	BBC3, CORO1A, HLA-DQB1, IFIT1B, Iigp1, PSMB9, Tgtp1/Tgtp2
		STAT6	transcription regulator	1.13×10^{-4}	1.98	EPHX1, GBP2, IFIT1B, Iigp1, NEDD1, PNPLA2, Tgtp1/Tgtp2
		ERK1/2****	group	3.19×10^{-4}	1.96	PSMB10, PSMB8, PSMB9, TAPBP, VCAM1
		IL17A****	cytokine	1.16×10^{-3}	-1.95*	GBP2, IFIT1B, Tgtp1/Tgtp2, VCAM1
		FOXO3**	transcription regulator	1.85×10^{-3}	2.07	AQP4, BBC3, PTPRC, VCAM1
		IL15****	cytokine	2.15×10^{-3}	-1.98*	BBC3, PSMB10, PSMB8, PSMB9
		ETV6-RUNX1****	fusion gene/product	1.70×10^{-2}	1.98*	CORO1A, GBP2, PSMB9, PTPRC
	^{177}Lu + A1M	STAT1	transcription regulator	1.29×10^{-7}	-2.01	BAD, Cyp2d9 (includes others), GBP2, IFIT1B, Iigp1, PSMB10, PSMB8, PSMB9, Tgtp1/Tgtp2
		TRIM24	transcription regulator	4.29×10^{-7}	2.45	GBP2, IFIT1B, Iigp1, PSMB10, PSMB8, PSMB9, Tgtp1/Tgtp2
		IL10RA	transmembrane receptor	3.49×10^{-5}	2.13	ARG2, GBP2, Iigp1, LUM, MEP1A, PSMB8, PSMB9, Tgtp1/Tgtp2
		IFNG	cytokine	6.36×10^{-5}	-2.32	ACE, AIF1, ARG2, BBC3, C1QB, GBP2, HLA-DQB1, Iigp1, PSMB10, PSMB8, PSMB9, Tgtp1/Tgtp2
		LHX1	transcription regulator	7.36×10^{-5}	-2.02	AADAT, Kap, MEP1A, MEP1B, SLC22A24
		SIRT1	transcription regulator	7.60×10^{-5}	2.50	BBC3, CORO1A, HLA-DQB1, HMGCR, IFIT1B, Iigp1, PSMB9, Tgtp1/Tgtp2
		PIK3CG**	kinase	4.97×10^{-4}	1.95	AIF1, GBP2, Iigp1, Tgtp1/Tgtp2
	A1M	STAT1	transcription regulator	6.86×10^{-7}	-2.43*	BAD, Cyp2d9 (includes others), GBP2, Iigp1, PSMB8, PSMB9, TAP1, Tgtp1/Tgtp2
		TRIM24	transcription regulator	7.59×10^{-7}	2.45*	GBP2, Iigp1, PSMB8, PSMB9, TAP1, Tgtp1/Tgtp2
		Ifnar	group	6.61×10^{-6}	-2.24*	GBP2, PSMB8, PSMB9, TAP1, TAPBP
		LHX1	transcription regulator	1.14×10^{-5}	-2.24*	AADAT, Kap, MEP1A, MEP1B, SLC22A24

		NRAS	enzyme	1.57×10^{-5}	2.24*	GBP2, Iigp1, PSMB8, TAP1, Tgtp1/Tgtp2
		IFNG	cytokine	7.36×10^{-6}	-3.23*	ACE, ARG2, GBP2, HLA-DQB1, Iigp1, PSMB8, PSMB9, SLC2A4, TAP1, TAPBP, Tgtp1/Tgtp2
		IGF1****	growth factor	2.38×10^{-4}	2.61	BAD, PSMB8, PSMB9, SLC2A4, TAP1
		SIRT1	transcription regulator	3.60×10^{-4}	2.41*	HLA-DQB1, HMGCR, Iigp1, PSMB9, TAP1, Tgtp1/Tgtp2
		mir-21	microrna	9.77×10^{-4}	2.00*	GBP2, Iigp1, TAP1, Tgtp1/Tgtp2
**7 d**	^{177}Lu	IL10RA****	transmembrane receptor	3.86×10^{-2}	2.01	CLIC6, IFI16, LTC4S, Tgtp1/Tgtp2
	^{177}Lu + A1M	CCNC	other	1.59×10^{-12}	2.02*	ACACA, CD36, FABP4, FASN, LIPE, PLIN1, PNPLA2, UCP1
		PEBP1****	other	2.23×10^{-8}	2.02	ACACA, Aldh1a7, FASN, PRKAR2B, RETN, UCP1
		MEDAG	other	6.50×10^{-8}	2.00*	CD36, FASN, LIPE, PLIN1
		N-cor	group	2.53×10^{-4}	-2.21*	ACACA, CD36, FABP4, FASN, PLIN1
		NOS2	enzyme	4.27×10^{-4}	-2.31	BAX, FABP4, FASN, KRT13, MB, Tgtp1/Tgtp2
		FST***	other	6.05×10^{-6}	2.14*	CD36, CPS1, FABP4, THRSP, UCP1
		CCR2	g-protein coupled receptor	3.77×10^{-4}	2.18*	ARG1, COL1A1, DCN, DPT, OGN
		mir-21	microrna	1.84×10^{-3}	2.68	AIF1, COL1A1, COL3A1, IGHM, Tgtp1/Tgtp2
		VEGFA****	growth factor	3.68×10^{-3}	1.98*	COL1A1, FABP4, HLA-DQB1, UCP1
		CD44	other	2.09×10^{-2}	2.00*	CD36, COL1A1, COL3A1, FASN
	A1M	MRTFB	transcription regulator	4.24×10^{-4}	-2.38	CMA1, LCN2, LTF, Ngp, S100A9
		MRTFA	transcription regulator	4.86×10^{-4}	-2.29	CMA1, LCN2, LTF, Ngp, S100A9
		CEBPA**	transcription regulator	5.87×10^{-3}	2.12	AKR1C3, LCN2, LTF, S100A9, SFTPB

Kidney cortex

Time	Treatment	Upstream Regulator	Molecule type	p-value	z-score	Target molecules in dataset
**24 h**	^{177}Lu	AR****	ligand-dependent nuclear receptor	4.09×10^{-2}	1.96*	BMPR2, CAST, CDKN1B, REN, SCEL, ZYX
		IFNG	cytokine	1.18×10^{-4}	-2.31*	AIF1, ALDH1A3, CD74, CYBB, ECE1, GBP2, HLA-DQA1, HLA-DQB1, PARVG, PPP1R1B, PSMB9, SDC4, SMAGP, Tgtp1/Tgtp2
		STAT1**	transcription regulator	1.40×10^{-4}	-2.18*	ALDH1A3, BAD, CAND2, GBP2, HLA-DQA1, PSMB9, SMAGP, Tgtp1/Tgtp2
		ETV6-RUNX1****	fusion gene/product	5.31×10^{-4}	2.43*	CORO1A, CYBB, GBP2, ITGB2, MGMT, PSMB9, PTPRC, STMN1
		SSB	enzyme	1.58×10^{-3}	2.00*	CD74, CYBB, HLA-DQA1, HLA-DQB1
		TRIM24**	transcription regulator	4.13×10^{-3}	1.98*	GBP2, MGMT, PSMB9, Tgtp1/Tgtp2

		NFkB (complex)****	complex	9.48 × 10-3	-2.22*	BAD, CD74, CYBB, HMOX1, PSMB9, SDC4
		mir-21****	microrna	1.27 × 10-2	2.00*	AIF1, BMPR2, GBP2, Tgtp1/Tgtp2
		TNF****	cytokine	3.22 × 10-2	-2.23*	ALDH1A3, CYBB, GBP2, HMOX1, MCM3, PSMB9, PTPRC, SDC4, Tgtp1/Tgtp2
	A1M	CEBPB***	transcription regulator	1.42 × 10-2	1.98*	Abcb1b, CDKN1B, MCM3, SAA1, ZYX
**7 d**	177Lu	NRAS	enzyme	8.81 × 10-4	-2.00*	BAX, EPHX1, KCTD12, PHLDA3
	A1M	KCNK9	ion channel	7.63 × 10-7	-2.20	CALB1, Gypa, HP, LCN2, NEFL
		MRTFB	transcription regulator	5.47 × 10-3	-1.99	CAMP, LCN2, Ngp, S100A9
		IL6***	cytokine	2.00 × 10-3	2.12	Chil3/Chil4, HP, Ighg2b, IL6ST, LCN2
		MRTFA	transcription regulator	6.08 × 10-3	-1.99	CAMP, LCN2, Ngp, S100A9

Bone marrow

Time	Treatment	Upstream Regulator	Molecule type	p-value	z-score	Target molecules in dataset
**24 h**	177Lu + A1M	SRF	transcription regulator	3.13 × 10-8	2.51	ACTA1, CKM, DES, FHL1, LDB3, MYH1, MYH7, MYL3, MYOM1, Nebl, Tpm2, TTN
		MYOD1**	transcription regulator	1.33 × 10-9	2.45*	ACTA1, ANKRD2, ATP2A1, CKM, DES, MYLPF, TNNC2, TNNT2
		KDM5A	transcription regulator	8.57 × 10-9	-3.16*	ACTN2, FXYD1, MYH7, MYH8, MYL6B, PGAM2, TNNC2, TNNT2, Tpm2, TRIM72
		MYOCD**	transcription regulator	1.82 × 10-8	2.76*	ACTA1, ACTN2, DES, MYH7, MYL2, TNNI1, TNNT2, TTN
		Bvht	other	2.25 × 10-8	2.81*	MYH7, MYL2, MYL3, MYOM1, SMYD1, TNNI1, TNNT2, TTN
		TBX5****	transcription regulator	4.66 × 10-7	2.37*	ACTN2, DES, MYL2, MYLPF, TNNT2, TTN
		RB1	transcription regulator	6.73 × 10-7	2.75	ACTN2, CKM, COL5A1, FXYD1, MECR, MYH7, MYH8, MYL6B, PGAM2, TNNC2, TNNT2, Tpm2, TRIM72
		SMTNL1	other	9.35 × 10-7	-2.24*	ACTA1, FLNC, MYOM1, TNNC2, Tpm2
		Gm15807/Hmgn5	other	1.56 × 10-6	2.24*	ACTA1, ATP2A1, MYL3, TNNC2, Tpm2
		FST***	other	1.02 × 10-5	2.20*	ATP1A2, COX7A1, FABP3, SAA1, SERPINA1
		HAND2****	transcription regulator	7.93 × 10-6	2.16*	ACTA1, ACTN2, DES, TNNT2, TTN
		NOS2	enzyme	1.30 × 10-5	-2.59	ACTA1, COX6A2, COX7A1, MB, MYH7, MYL2, MYL3, TNNT2
		STK40***	kinase	2.44 × 10-4	2.00*	ACTA1, CKM, DES, MYH8
		KMT2D****	transcription regulator	3.71 × 10-2	1.98*	FABP3, FHL1, MYOM3, TNNT2
		GATA4**	transcription regulator	2.08 × 10-4	2.25	ACTA1, ACTN2, DES, MYH7, TNNT2, TTN
		STAT5A	transcription regulator	2.29 × 10-2	-2.26	MYH1, MYH7, MYL2, TNNI1, TPM3
	A1M	SMTNL1	other	1.44 × 10-5	2.23	MYH4, TNNC2, TNNI2, TNNT3
		MYOD1**	transcription regulator	2.84 × 10-10	-2.94	ANKRD2, MYH3, MYH4, MYLPF, TNNC2, TNNI2, TNNT2, TNNT3

		KDM5A	transcription regulator	5.59×10^{-5}	2.12	Actn3, FXYD1, MYH4, TNNC2, TNNI2, TNNT2
		SMARCA4****	transcription regulator	4.67×10^{-4}	-2.66	HCLS1, LUM, MTRES1, MYH3, MYLPF, POLE4, TNNC2, TNNI2, TNNT2, TNNT3
		DNMT3B	enzyme	7.81×10^{-3}	2.20	CASQ1, RYR2, TNNT2, TNNT3
**7 d**	^{177}Lu	DNMT3B	enzyme	1.04×10^{-5}	2.45*	MYH7, MYH7B, MYL2, MYL3, TNNI1, TNNT2
		MYOCD****	transcription regulator	4.20×10^{-6}	-2.18*	ACTN2, MYH7, MYL2, TNNI1, TNNT2
		Bvht	other	4.80×10^{-6}	-2.22*	MYH7, MYL2, MYL3, TNNI1, TNNT2
		DNMT3A	enzyme	1.25×10^{-5}	2.01	MYH7, MYH7B, MYL2, MYL3, TNNI1, TNNT2
		NOS2***	enzyme	3.50×10^{-4}	2.05	KRT13, MYH7, MYL2, MYL3, TNNT2
		KDM5A	transcription regulator	8.66×10^{-4}	2.00*	ACTN2, MYH7, MYL6B, TNNT2
	^{177}Lu + A1M	SMTNL1	other	1.10×10^{-15}	-3.36	ACTA1, FLNC, MYH4, MYL1, MYOM1, PYGM, TNNC2, TNNI2, TNNT3, Tpm1, Tpm2
		MYOD1**	transcription regulator	4.39×10^{-17}	2.79	ACTA1, ATP2A1, DES, DMD, ENO3, INPP5K, MYH3, MYH4, MYL1, MYLPF, TNNC2, TNNI2, TNNT2, TNNT3
		KDM5A	transcription regulator	1.91×10^{-11}	-2.60	ACTC1, Actn3, MFN2, MYH4, MYH8, MYL1, PGAM2, RYR1, TNNC2, TNNI2, TNNT2, Tpm1, Tpm2, TRIM72
		RB1	transcription regulator	8.07×10^{-12}	2.72	ACTC1, Actn3, BAK1, BCL2L11, Esrra, Krt10, KRT5, LOXL2, MFN2, MYH4, MYH8, MYL1, PGAM2, RYR1, TNNC2, TNNI2, TNNT2, Tpm1, Tpm2, TRIM72, TUBG1, ZNF638
		SRF	transcription regulator	6.85×10^{-9}	2.91	ACTA1, ACTC1, BCL2L11, DES, DMD, LDB3, MYH1, MYH4, MYL1, MYOM1, Nebl, Tpm1, Tpm2, TTN, TUBB4B
		PTCH1	transmembrane receptor	3.11×10^{-6}	-2.27	ACTA1, MYH1, TNNI2, TNNT3, Tpm2
		FOXO1**	transcription regulator	1.48×10^{-2}	-2.13	BANK1, BCL2L11, CCR7, CD79B, CDKN2C, CKMT2, GZMA, MB
		HDAC4	transcription regulator	3.78×10^{-3}	-2.00	CACNA2D1, DMD, MYBPC2, MYOT, MYOZ1
	A1M	SMTNL1	other	6.10×10^{-14}	-4.08	ACTA1, FLNC, MYH4, MYL1, MYOM1, PYGM, TNNC2, TNNI2, TNNT3, Tpm1, Tpm2
		MYOD1**	transcription regulator	4.63×10^{-12}	2.53*	ACTA1, ATP2A1, DES, ENO3, MYH3, MYH4, MYL1, MYLPF, TNNC2, TNNI2, TNNT2, TNNT3
		KDM5A	transcription regulator	2.31×10^{-9}	-2.52*	ACTC1, Actn3, MFN2, MYH4, MYH8, MYL1, PGAM2, RYR1, TNNC2, TNNI2, TNNT2, Tpm1, Tpm2, TRIM72
		DMD	other	5.96×10^{-9}	1.96	Actn3, AMPD1, ATP2A1, DES, LDB3, LYZ, MYBPC2, MYH1, MYH4, MYH8, MYL1, MYOZ1, OBSCN, PYGM, SERPINA1, TMOD4, TNRC6B

	SRF	transcription regulator	8.05×10^{-7}	3.58	ACTA1, ACTC1, AKAP12, DES, Igkv1-117, LDB3, MYH1, MYH4, MYL1, MYOM1, Nebl, Tpm1, Tpm2, TTN, TUBB4B
	RB1	transcription regulator	8.66×10^{-7}	3.04	ACTC1, Actn3, BAK1, Krt10, LOXL2, MFN2, MYH4, MYH8, MYL1, PGAM2, RYR1, SAFB, TNNC2, TNNI2, TNNT2, Tpm1, Tpm2, TRIM72, ZNF638
	NOS2**	enzyme	1.18×10^{-4}	-1.99	ACTA1, ACTC1, CD3E, COX6A2, IGHG1, KRT13, MB, MYL2, TNNT2, TNNT3
	HSP90B1****	other	7.89×10^{-4}	-2.24*	RPLP2, SNX3, TOMM20, VAMP2, VAMP3
	mir-122****	microrna	3.23×10^{-2}	2.00*	MAP4, SERPINB6, TPD52L2, VAMP3
	MITF****	transcription regulator	3.42×10^{-2}	-2.22*	CDCA3, COL2A1, FMOD, SNW1, SOX6

*No bias correction of the z-score was made

**|z-value| >2 not fulfilled when considering molecules and/or relationships in mouse only

***|z-value| >2 only fulfilled when considering molecules and/or relationships in mouse only

****Not found when considering molecules and/or relationships in mouse only

Supplemental Table S5. *In silico* toxicity functions related to hepatotoxicity or cardiovascular toxicity identified by IPA using expression data of differentially regulated proteins (|FC| ≥ 1.5). Data is given for kidney cortex, kidney medulla and bone marrow at 24 hours and 7 days after treatment with ^{177}Lu-octreotate (^{177}Lu), ^{177}Lu-octreotate with A1M (^{177}Lu + A1M) or with A1M alone. Bias corrected Z-score predicts activations state, i.e. z ≤ -2.0 indicates inhibition and z ≥2.0 indicates activation.

Kidney Cortex

Time	Treatment	Category	Function	p-value	z-score	Target proteins in data set
7 d	^{177}Lu	Liver Necrosis/Cell Death	necrosis	3.03×10^{-2}	1.89	USP2, GIMAP1-GIMAP5, SOD1, BAX
7 d	^{177}Lu + A1M	Liver Steatosis	hepatic steatosis	2.30×10^{-5}	0.86	CD36, CAV1, FABP4, PRKAR2B, PNPLA2, SOD1, LIPE, FASN, MAT1A, ACACA, SIRT1
7 d	^{177}Lu + A1M	Heart Failure	failure	1.09×10^{-3}	-1.09*	MB, CD36, CAV1, PNPLA2, CKM, CA3, AOC3, SIRT1
7 d	^{177}Lu + A1M	Cardiac Fibrosis	fibrosis	1.02×10^{-2}	0.01	POSTN, MB, CAV1, PNPLA2, IGHM
7 d	A1M	Liver Steatosis**	hepatic steatosis	2.08×10^{-2}	1.20	PNPLA2, SOD1, GBP2, LCN2, FOXO3
7 d	A1M	Liver Necrosis/Cell Death	necrosis	2.17×10^{-2}	1.18	ATF2, USP2, SOD1, FOXO3

Kidney medulla

Time	Treatment	Category	Function	p-value	z-score	Target proteins in data set
24 h	^{177}Lu	Liver Necrosis/ Cell Death	necrosis	5.42×10^{-3}	-0.89	CDKN1B, SOD1, ITGB2, PTPRC, HMOX1, BAD
24 h	^{177}Lu	Liver Inflammation/Hepatitis**	inflammation	5.60×10^{-3}	-0.47	AMY2B, IL10RB, HLA-DQA1, SOD1, GBP2, ITGB2, PTPRC, HMOX1
24 h	^{177}Lu	Liver Necrosis/ Cell Death	cell death	8.67×10^{-3}	-1.04	CDKN1B, ITGB2, PTPRC, HMOX1, BAD
24 h	^{177}Lu	Cardiac Necrosis/ Cell Death	cell death	1.28×10^{-2}	-0.22	ZYX, CDKN1B, CYBB, HMOX1
24 h	^{177}Lu	Renal Necrosis/ Cell Death	cell death	3.17×10^{-2}	-0.81	MAVS, CDKN1B, SOD1, CYBB, BAD, STMN1
24 h	^{177}Lu	Liver Steatosis**	hepatic steatosis	3.20×10^{-2}	0.51	CDKN1B, HLA-DQA1, SOD1, RGCC, GBP2, HMOX1
24 h	^{177}Lu+A1M	Liver Steatosis	hepatic steatosis	1.56×10^{-2}	-0.58	CDKN1B, Akr1b7, NUCKS1, SOD1, RGCC, DGAT1
24 h	^{177}Lu+A1M	Liver Damage	damage	1.97×10^{-2}	2.12	SAA1, SOD1, SDC4, PAWR
24 h	^{177}Lu+A1M	Liver Necrosis/ Cell Death**	necrosis	4.70×10^{-2}	0.36	SAA1, CDKN1B, SOD1, ABL2

24 h	A1M	Liver Damage	damage	3.27×10^{-2}	2.19	SAA1, SOD1, SDC4, PAWR

Bone marrow

Time	Treatment	Category	Function	p-value	z-score	Target proteins in data set
24 h	^{177}Lu+A1M	Heart Failure	failure	4.08×10^{-7}	-1.40*	MYH7, CRYAB, MYL2, TNNT2, DES, ATP1A2, MYLK3, JPH2, MYOM1, MB, CKM, Mff, TTN
7 d	^{177}Lu+A1M	Heart Failure	failure	5.92×10^{-4}	-1.14	MB, AMY2B, CACNA2D1, TNNT2, DES, ATP1A2, Mff, TTN, JPH2, RYR2, MYOM1
7 d	^{177}Lu+A1M	Liver Hyperplasia/ Hyperproli-feration**	liver tumor	7.59×10^{-3}	0.74	ZNF638, UTP20, TUBG1, OBSCN, DES, EEF1A2, TUBA1C, TEX2, JPH2, USP1, MYH3, MFN2, ZNF318, ZNF787, AMPD1, RYR1, TSC2, PIP5K1A, FLNC, CKS2, MYOT, DMD, SPRYD4, ERCC3, TMEM63A, PAG1, MYH1, ARHGAP10, TIPRL, ERLIN2, FPR2, KAT5, CPT1B, TUBB4B, RSL24D1, RPP40, GTPBP3, EIF2AK4, SLC38A2, CACNA2D1, ACTA1, KRT16, POLL, NDRG2, PYGM, ATE1, BCL2L11, ACTC1, EHMT2, Esrra, API5, ATP1A2, RGS3, MYOM1, TUBB2A, ATP13A3, MYH4, CDCA2, ZNF280D, MTRF1L, KRT71, ARHGEF18, MPP7, LAMA2, TBC1D2, DDX10, AMY2B, TBCEL, TUBB, TAF2, KRI1, TNNT2, TMEM230, BCHE, ATAD1, NOA1, CERS2, CKMT2, MYLPF, LDB3, MYH8, TTN, BANK1, RYR2, MINK1
7 d	^{177}Lu+A1M	Liver Hyperplasia/ Hyperproli-feration**	developme nt	3.84×10^{-2}	0.73	NDRG2, TSC2, Esrra, CERS2
7 d	A1M	Heart Failure	failure	1.22×10^{-3}	-0.93*	AMY2B, MYL2, TNNT2, DES, FADD, ATP1A2, MYLK3, JPH2, MYOM1, MB, Mff, TTN, RYR2

7 d	A1M	Increased Levels of Hematocrit**	hematocrit **	3.98×10^{-2}	-1.96*	DMTN, KMT2E, ADD2, ZFP36L2

*No bias correction of the z-score was made
**Not found when considering molecules and/or relationships in mouse only
